# Endothelial epidermal growth factor receptor is of minor importance for vascular and renal function and obesity-induced dysfunction in mice

**DOI:** 10.1038/s41598-021-86587-3

**Published:** 2021-03-31

**Authors:** Barbara Schreier, Christian Stern, Virginie Dubourg, Alexander Nolze, Sindy Rabe, Sigrid Mildenberger, Claudia Wickenhauser, Michael Gekle

**Affiliations:** 1 Julius-Bernstein-Institute of Physiology, Martin Luther University Halle-Wittenberg, Magdeburger Strasse 6, 06112 Halle (Saale), Germany; 2 Institute of Pathology, Martin Luther University Halle-Wittenberg, Halle, Germany

**Keywords:** Cardiovascular biology, Medical research, Preclinical research, Obesity

## Abstract

Vascular EGF receptors (EGFR) influence function and structure of arterial vessels. In genetic mouse models we described the role of vascular smooth muscle (VSMC) EGFR for proper physiological function and structure as well as for pathophysiological alterations by obesity or angiotensin II. As the importance of endothelial (EC) EGFR in vivo is unknown, we analyzed the impact of EC-EGFR knockout in a conditional mouse model on vascular and renal function under control condition as well as in obesity and in comparison to VSMC-KO. Heart and lung weight, blood pressure and aortic transcriptome (determined by RNA-seq) were not affected by EC-EGFR-KO. Aortic reactivity to α1-adrenergic stimulation was not affected by EC-EGFR-KO contrary to VSMC-EGFR-KO. Endothelial-induced relaxation was reduced in abdominal aorta of EC-EGFR-KO animals, whereas it was enhanced in VSMC-EGFR-KO animals. Mesenteric arteries of EC-EGFR-KO animals showed enhanced sensitivity to α1-adrenergic stimulation, whereas endothelial-induced relaxation and vessel morphology were not affected. Renal weight, histomorphology, function (albumin excretion, serum creatinine, fractional water excretion) or transcriptome were not affected by EC-EGFR-KO, likewise in VSMC-EGFR-KO. High fat diet (HFD) over 18 weeks induced arterial wall thickening, renal weight increase, creatininemia, renal and aortic transcriptome alterations with a similar pattern in EC-EGFR-WT and EC-EGFR-KO animals by contrast to the previously reported impact of VSMC-EGFR-KO. HFD induced endothelial dysfunction in abdominal aortae of EC-EGFR-WT, which was not additive to the EC-EGFR-KO-induced endothelial dysfunction. As shown before, VSMC-EGFR-KO prevented HFD-induced endothelial dysfunction. HFD-induced albuminuria was less pronounced in EC-EGFR-KO animals and abrogated in VSMC-EGFR-KO animals. Our results indicate that EC-EGFR, in comparison to VSMC-EGFR, is of minor and opposite importance for basal renovascular function as well as for high fat diet-induced vascular remodeling and renal end organ damage.

## Introduction

The epidermal growth factor receptor (EGFR/ErbB1), a member of the ErbB-receptor tyrosine kinase family, is activated by binding e.g. epidermal growth factor (EGF) or heparin bound EGF (HB-EGF) and modulates signaling pathways that affect cell differentiation, migration and matrix homeostasis^1^. EGFR can also be transactivated by receptors for vasoactive substances, thereby contributing to vascular tone, dysfunction and remodeling^2,3^ as a heterologous transducer for non-EGFR ligands.

The relevance of vascular smooth muscle cells (VSMC)-EGFR for basal vascular function, for angiotensin II (AII)- or obesity-induced structural and functional vascular remodeling, as well as for complete renal end organ damage succeeding vascular remodeling, was shown^4,5^. These and other studies document the importance of VSMC-EGFR in cardiovascular health and disease^3,6–10^. In addition, systemic inhibitors of EGFR-kinase activity improved vascular function and vascular wall homeostasis in diabetic animals^7–13^. Thus, vascular EGFR could not only be of pathogenetic but also of therapeutic cardiovascular importance when conventional therapies fail, having pharmacological tools already at hands. To evaluate this concept further, the mechanistic role of vascular EGFR should be understood, meaning that the respective relevance of VSMC- and endothelial cell (EC)- EGFR needs to be determined.

Hitherto, the importance of endothelial EGFR is not as well understood as the one of VSMC. However, there is evidence from pharmacological studies for a modulatory role of EGFR in normal tissue, besides its enhanced expression in tumor endothelium^14^ or during the development of endothelial dysfunction^3^. But, because these studies could not differentiate between VSMC- and EC-EGFR, investigations in a conditional KO mouse model are required.

Recently, we established a knockout (KO) mouse model with deletion of the EGFR in VSMC^15^ that confirmed the involvement of VSMC-EGFR in basal blood pressure homeostasis, ageing-related vascular changes, acute AII vascular responsiveness^15^ and pathological AII action in vivo^4^. Furthermore, this model unveiled the importance of VSMC-EGFR for obesity-induced vascular and renal damage^5^. The present study investigates in vivo and ex vivo the role of EC-EGFR under basal conditions as well as during high fat diet-induced obesity/diabetes mellitus type 2 (DMT2) using the (B6.Cg-Tg(Tek-cre)1Ywa/J = Tie2^CRE^)^16^ mouse model to generate EGFR deletion, with respect to vascular remodeling, vascular gene expression and renal damage. Furthermore, we compare the possible importance of EC-EGFR with the previously investigated role of VSMC-EGFR, assuming that EC-EGFR is of less importance. The data presented confirm this hypothesis under basal as well as under pathological condition.

## Materials and methods

All mouse experiments were approved by the local government (Landesverwaltungsamt Sachsen-Anhalt, Germany, Az.: 505.6.3-42502-2-1389 MLU_G; Veterinäramt Stadt Halle, Germany; Bescheid T16/2019) and conducted in accordance with the National Institutes of Health Guide for the Care and Use of Laboratory Animals, the ARRIVE guidelines and under consideration of the 3R-principle.

### Animals

As already described before^4,5,15^ mice were kept at constant temperature of 22 ± 2 °C, relative humidity of 30–60%, under a 12/12 h light–dark cycle with ad libitum access to water and standard chow. Knock-out in endothelial cells was achieved by mating *EGFR*^*flox/flox*^ C57BL/6 mice (originally provided by Maria Sibilia, Vienna, Austria) with B6.Cg-Tg(Tek-cre)1Ywa animals^16^ from Jackson Laboratory (Stock No: 008863, C57BL/6J genetic background). Inducible knock out for EGFR in VSMC was created by mating *EGFR*^*flox/flox*^ C57BL/6 mice (originally provided by Maria Sibilia, Vienna, Austria) with B6.FVB-Tg(Myh11-cre/ERT2)1Soff mice (= *iSMMHC-Cre*^+*/−*^ C57BL/6N mice, Jackson laboratory, stock no: 019079, C57BL/6N genetic background, originally provided by Stefan Offermanns, Bad Nauheim, Germany)^4,5,15^. As controls (wild type, WT) mice without LoxP-sites but carrying the Cre-recombinase under control of the *iSMMHC*-promotor and treated with tamoxifen (= VSMC-model) or carrying the Cre-recombinase under control of the *Tie2*-promotor (= EC-model) were employed. HFD (60% fat share of energy versus 10% in SFD) was started at 6 weeks of age. Genotyping was performed on tail biopsies by PCR as previously described^2^. Knockout was confirmed by determination of the excision of EGFR exon 1 by genomic PCR. Successful excision of the floxed DNA-fragment in the EGFR gene, comprising exon 1, was determined with DNA extracted from lung or aortic tissue applying the following protocol: denaturation at 94 °C for 3 min followed by 35 cycles with denaturation at 94 °C for 30 s, annealing at 66,1 °C for 45 s and extension at 72 °C for 4 min. Final extension was performed at 72 °C for 4.5 min. The reaction mix contained additional 2 mM Mg_2_Cl. Forward primer: GGGTGACGTGTTCCCATTCA, reverse primer: AAAGTTTGCTACCGGCCTCA. Product length without excision = 2579 bp, product length with excision = 549 bp. DNA was extracted by proteinase K digestion and salt precipitation. PCR products were analysed by agarose gel electrophoresis followed by iodidium bromide staining (Supplementary methods SM01). To confirm reduced EGFR mRNA expression we performed quantitative RT-PCR (qRT-PCR) in lung tissue. RNA was isolated using InviTrap spin tissue RNA mini kit (Invitek Molecular GmbH) following manufacturer’s instructions. DNA contamination was removed (DNAse I, New England Biolabs) and reverse transcription (RT) was performed using random primers and Super-Script II reverse transcriptase (Invitrogen, Life Technologies) according to manufacturer’s instructions. 1 µL of the obtained cDNA was used in RT-qPCR (AriaMx Real-Time PCR System, Agilent Technologies). EGFR forward primer: GACCTTCACATCCTGCCAGT. EGFR reverse primer: GCATGGAGGTCAGTCCAGTT. qPCR efficiency was > 90%. The relative mRNA expression of the genes of interest was calculated according to the 2^-ΔΔCt^ method, using the 18S RNA signal for normalization. 18S forward primer: GTAACCCGTTGAACCCCATT. 18S reverse primer: CCATCCAATCGGTAGTAGCG. Each sample was analyzed as triplicate. Results are shown in Supplementary methods SM02, expressed as mean difference between wild-type and knock out ± standard error of mean. Breeding of the animals and assignment to experimental groups was performed randomly by place holder numbers before knowledge regarding the animals was obtained. During further experimentation the genotype of the animals was blinded by pseudonymisation (assignment of numbers). Due to the differences in weight gain, the type of diet could not be blinded.

For body and organ weight analysis as well as for blood pressure measurements by Millar catheter of the VSMC-model new animal cohorts (WT-SFD, WT-HFD, KO-SFD, KO-HFD) were generated. For the aortic ring force development split into thoracic and abdominal aorta a separated reanalysis of thoracic and abdominal aortic rings investigated previously was performed^5^.

### EGFR ELISA

In order to obtain an initial indication regarding the relative expression of EGFR in endothelial cells compared to vascular smooth muscle cells, we quantified EGFR protein in primary cells of human origin (Supplementary methods SM03). For additional referencing we included primary human mesangial cells (smooth muscle like cell type), renal proximal tubule cells (epithelial cell type) and HEK293 cell. EGFR expression was determined by an EGFR sandwich ELISA (R&D Systems, Minneapolis, MN) according to the manufacturer’s protocol. Cells were lysed at 4 °C in extraction buffer [10 mM Tris (pH 7.4), 100 mM NaCl, 1 mM EDTA, 1 mM EGTA, 1 mM NaF, 20 mM Na4P2O7, 2 mM Na3VO4, 1%Triton X-100, 10%glycerol, 0.1%SDS, 0.5%deoxycholate, protease inhibitor cocktail (1:1000)]. Protein content was determined using the BCA reagent from Pierce (Rockford, IL). SM03 shows that EGFR expression was lowest, but clearly distinct from zero, in endothelial cells.

### Invasive measurement of blood pressure

Intravasal blood pressure measurements (diastolic, systolic, mean) were performed in anaesthetized (80 mg/kg body weight (BW) ketamine and 120 mg/kg BW xylazine, Sigma-Aldrich, St. Louis, USA) mice as described before^15^. The right jugular vein was cannulated for infusion of 2% bovine serum albumin (Sigma-Aldrich, Steinheim, Germany) in Ringer lactate solution (for 1 L: NaCl 5.9 g, KCl 0.3 g, CaCl2 0.22 g, Na-Lactate 2.8 g) at 4 µl/g BW/min. A Millar catheter (size 1F, Millar Instruments, Houston, USA) was inserted into the right carotid artery. After a 20-min stabilization period systolic, diastolic and mean blood pressure were measured (PowerLab data acquisition systems, Spechbach, Germany) and pulse pressure was calculated with the LabChart7 software (ADI instruments, Spechbach, Germany). To analyze the impact of volume load on blood pressure a bolus of 50 µl Ringer Lactate with a subsequent infusion of 100 µl Ringer lactate solution (within one min, infusion rate 6 ml/h) was infused via the jugular vein catheter and the blood pressure was analyzed for 10 min. Subsequently, to analyze the reactivity of blood pressure upon phenylephrine (PE), a bolus of 100 µg PE / kg BW was given followed by an infusion of 100 µl Ringer lactate solution as described above within a minute (infusion rate: 6 ml/h). The change in blood pressure was analyzed for a subsequent 20 min time period.

### Measurement of aortic ring force

Wire myography was performed using a system of DMT (Aarhus, Denmark), according to the protocols published before^4,5,15^. Aortic rings were equilibrated in modified aerated Krebs–Ringer solution (20% O_2_, 5% CO_2_) at 37 °C for 30 min. At the beginning and the end of the equilibration the physiological salt solution was changed once, followed by the application of a strain resulting in a force of 12mN^4,15^. This strain resulted in a similar change in vessel circumference (dL) and similar effective pressure values in both genotypes and was applied for 10 min prior to the first substance application. Wall stress and effective pressure were calculated as described by Mulvany and Halpern^17^. After each measurement, the chambers were flushed five times with Krebs–Ringer-solution, achieving an approximately 100,000 fold dilution of the substance, before a new reagent was tested. This did not apply for the relaxants carbamoylcholine chloride (carbachol) and S-Nitroso-*N*-acetyl-DL-penicillamine (SNAP). These substances were given at the point of stable force development of the previously administered vasoconstrictor.

### Pressurized mesenteric arteries

Pressure myography was performed using a system of DMT (Aarhus, Denmark)^4^. After mice were killed, the mesenteric bed was removed and transferred to cold (4 °C) physiological salt solution. Mesenteric (third or fourth order) arteries were mounted on glass cannulas to allow perfusion at physiological pressures (inlet pressure 60 mmHg, outlet pressure 45 mmHg). Vessels were superfused continuously with Krebs–Henseleit solution (20% O_2_, 5% CO_2_; pH, 7.4; 37 °C) composed of (mmol/l): 119 NaCl, 4.7 KCl, 25 NaHCO_3_, 1.2 KH_2_PO_4_, 1.6 CaCl_2_, 1.2 MgSO_4_, 0.03 EDTA and 11.1 glucose. The wake-up procedure was carried out according to the manufacturer’s instructions by stepwise pressurizing to 20, 40, 60, 80, or 100 mm Hg using servo control system. Diameter of the vessels was measured with a video microscope (Zeiss Axiovert, Oberkochen, Germany) and a data acquisition and analysis system (Danish Myo Technology A/S, DK-8382 Hinnerup, Denmark). A 60-mmol/l KCl challenge was performed after 45 to 60 min equilibration and before any other interventions.

### Harvesting of organs

Mice were sacrificed by cervical dislocation. Livers, kidneys, lungs, hearts and aortas were excised, carefully freed from adjacent tissues, and weighed. Tibia length was measured for normalization of organ weights. Parts of the tissues were immediately snap frozen in liquid nitrogen while parts were fixed in 5% paraformaldehyde solution. Tissues were dehydrated in increasing concentrations of methanol or isopropanol. After embedding in paraffin, 4 µm sections were cut^4^.

### Histomorphometric analysis

Morphometric analysis was performed as described before^2,4^ in a blinded way (pseudonymisation by number assignment). Sections were double-blinded investigated. Glomerular damage was assessed as Bowman and glomerular area, glomerular cellularity (nuclei per glomerulus) and glomerulosclerosis in kidney sections stained with periodic acid-Schiff’s reagent. At least 30 glomeruli per kidney were evaluated, and the given values are the mean per animal. The degree of glomerulosclerosis was determined using a semiquantitative scoring method^18^. Glomeruli were selected randomly and scored as follows: grade 0, normal; grade 1, sclerotic area 25% of total glomerular area; grade 2, sclerotic area 25%-50%; grade 3, sclerotic area 50%-75%; grade 4, sclerotic area 75%-100%. For media-to-lumen ratio media thickness was measured at ten different locations within the vessel wall and divided by the internal circumference of the aorta according to Liang^19^. Fibrosis was analyzed by staining with Sirius red followed by quantitative microscopic determination of the relative fibrotic area.

### Next generation sequencing and gene enrichment analysis

Total RNA was isolated as described^20^. Paired-end sequencing (2 × 150 bp) was performed with a HiSeq Illumina System by Novogene (UK) Co., Ltd (Cambridge, UK) in the same way as in our previous study^5^. Libraries were prepared with indexed adapters. Quality control was performed on the data provided by the service company (adaptors already clipped, fastQC, v0.11.3, https://www.bioinformatics.babraham.ac.uk/projects/fastqc/). Read mapping was done with Tophat2 (2.0.14)^21^ (mouse genome GRCm38/mm10) and counting was done with featureCounts (1.4.6)^22^. Genes were annotated with BiomaRt v93 (R package v2.36.1)^23^. Normalization and differential expression analysis were performed using R package EdgeR (3.20.8)^24^ from Bioconductor (https://www.bioconductor.org/). The counts were normalized using the “trimmed mean of M values” (TMM) method. A false discovery rate (FDR) of 0.05 was used to determine if genes were significantly regulated. Additional filters such as FPM > 5 and Fold Change > 1.5 were applied. Raw data are publicly available on the GEO (Gene Expression Omnibus) database (https://www.ncbi.nlm.nih.gov/geo). GEO IDs: GSE144838 for the VSMC model and GSE158197 for the EC model.

Gene ontology enrichment analysis was performed with g:Profiler (http://biit.cs.ut.ee/gprofiler/;^25^) and Ingenuity Pathway Analysis (IPA) software (Qiagen, Hilden, Germany) was used for functional analysis (including Canonical Pathways, Upstream Regulator and Downstream Effects Analyses; that are features not included in g:Profiler) on the lists of regulated genes (results of the differential expression analyses). Their Ensembl identifiers were mapped to networks available in the software database. For the canonical pathway analysis, enriched pathways were ranked according to how relevant they were for the genes provided as input. Multiple testing was performed using the Benjamini–Hochberg (B–H) procedure. Analyses were corrected for multiple testing as described for the corresponding tools.

### Determination of albumin, creatinine and glucose

Glucose consumption was assessed by commercially available kits (Glucose (HK) assay kit, Sigma, Darmstadt, Germany) according to the manufacturer’s instructions^26^. Albumin was determined by ELISA (Bethyl Laboratories, Montgomery, Texas, USA). Creatinine was determined enzymatically by the creatinase method (Diazyme, Dresden, Germany).

### Statistics

Data are presented as mean ± standard error of mean. As described before^5^ 2-way ANOVA (SFD vs HFD and WT vs KO) was performed, followed by post hoc testing. Student´s T-Test or Mann–Whitney Rank Sum Test were used as applicable according to pre-test data analysis by SigmaPlot 12.5. A p-value < 0.05 was considered significant. N = number of animals or vessels. Biometrical planning was performed under consideration of the 3R-principle with α = 0.05 and β = 0.8. Data from all experiments that proceeded technically according to plan were included into the analyses.

### Ethcs approval and consent to participate

All mouse experiments were approved by the local government (Landesverwaltungsamt Sachsen-Anhalt, Germany, Az.: 505.6.3-42502-2-1389 MLU_G; Veterinäramt Stadt Halle, Germany; Bes-cheid T16/2019) and conducted in accordance with the National Institutes of Health Guide for the Care and Use of Laboratory Animals, the ARRIVE guidelines and under consideration of the 3R-principle.

## Results

### Systemic parameters

**Figure 1:**
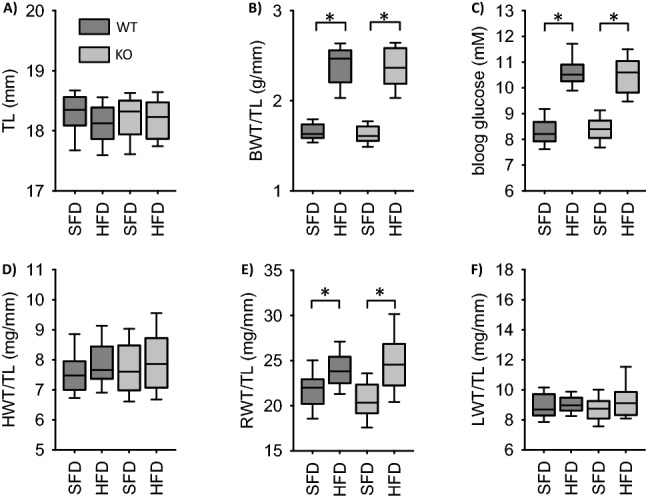
Influence of high fat diet on body weight (**A**,**B**), blood glucose (**C**) and heart (**D**), kidney (**E**) and lung (**F**) weight in EC-EGFR-WT and EC-EGFR-KO animals. Number of animals of the EC-model: N(WT, SFD) = 29, N(WT, HFD) = 30, N(KO, SFD) = 38, N(KO, HFD) = 39. Number of animals of the VSMC-model: N(WT, SFD) = 44, N(WT, HFD) = 38, N(KO, SFD) = 58, N(KO, HFD) = 54.*p < 0.05. *SFD* standard diet, *HFD* high fat diet, *BWT* body weight, *TL* tibia length, *HWT* heart weight, *RWT* renal weight, *LWT* lung weight, *VSMC* vascular smooth muscle cell.

**Figure 2:**
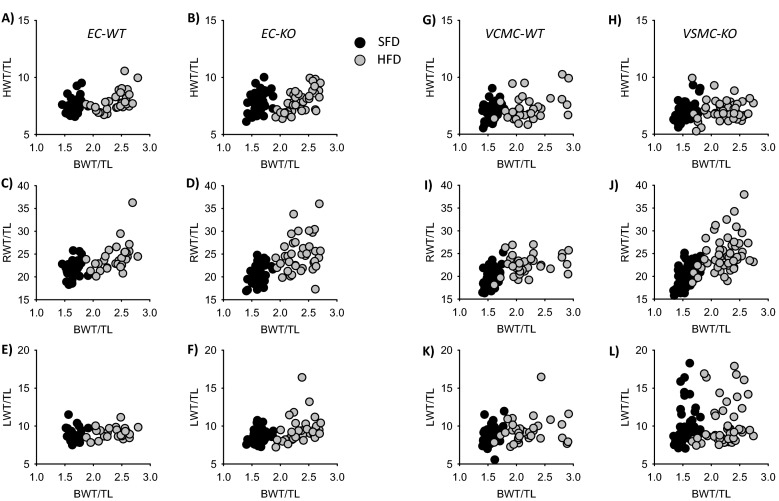
Scatter plots showing the correlation between body weight (BWT) and heart (HWT; **A**,**B**,**G**,**H**), kidney (RWT; **C**,**D**,**I**,**J**) or lung (LWT; **E**,**F**,**K**,**L**) weights in the four genotypes under standard fat diet (SFD) and high fat diet (HFD). TL = tibia length. Number of animals for the EC-model: N(WT, SFD) = 29, N(WT, HFD) = 30, N(KO, SFD) = 38, N(KO, HFD) = 39. Number of animals for the VSMC-model: N(WT, SFD) = 44, N(WT, HFD) = 38, N(KO, SFD) = 58, N(KO, HFD) = 54.

EC-EGFR-KO animals were born at a mendelian ratio and showed no gross abnormalities. There was no difference in body weight, tibia length or blood glucose compared to their wildtype littermates as well as to the VSMC-EGFR-WT animals (Fig. 1A–C, Supplementary Fig. SF01), except the known slightly higher blood glucose concentration^27^ under standard fat diet (SFD), most probably attributable to the genetic background. High fat diet (HFD) led to a similar increase in body weight and blood glucose in EC-EGFR-WT, EC-EGFR-KO, VSMC-EGFR-WT and VSMC-EGFR-KO (Fig. 1a–C, Supplementary Fig. SF01). Analysis of organ weights (Fig. 1A, Supplementary Fig. SF01) revealed no differences between the genotypes under control conditions for heart and kidney. There was a slight trend towards higher heart and kidney weights in EC-EGFR-WT compared to VSMC-EGFR-WT. HFD induced no change in heart weight but an increase in renal weight (normalized to tibia length (TL) or heart weight) that was aggravated in VSMC-EGFR-KO (EC: Fig. 1D,F, VSMC: Supplementary Fig. SF01). Scattered analysis shows that a susceptible subgroup of animals with substantially enhanced renal weight (> 25 mg/mm TL) was responsible for this effect (Fig. 2). This effect was much less pronounced in EC-EGFR-KO animals (Fig. 2, Supplementary Fig. SF02). Lung weight was not affected neither by genotype nor HFD in the EC-EGFR model (Fig. 2, Supplementary Figs. SF02 and SF03). By contrast, VSMC-EGFR-KO induced an increase in lung weight in a susceptible subgroup under SFD. HFD did not aggravate this effect (Fig. 2, Supplementary Fig. SF03).

By contrast to VSMC-EGFR-KO^15^, blood pressure was not significantly different between EC-EGFR-KO and EC-EGFR-WT under SFD (Supplementary Fig. SF04). HFD induced a slight increase in systolic and mean blood pressure in EC-EGFR-WT and EC-EGFR-KO animals.

### Functional vascular parameters

Isometric force measurements in aortic rings (conductance vessel) revealed no difference of action for the α1-adrenergic agonist, phenylephrine, in rings from EC-EGFR-KO as compared to EC-EGFR-WT animals under SFD (Supplementary Fig. SF05), in contrast to VSMC-EGFR-KO, as shown previously^5^. Reduction in phenylephrine induced-force generation during HFD was not prevented by EC-EGFR-KO.

HFD and EGFR-KO reduced KCl-induced contraction in the thoracic aorta from the VSMC-EGFR model but not in abdominal or thoracic aortae from EC-EGFR animals (Supplementary Fig. SF06). In the abdominal aorta, the U46619-induced (thromboxane analogue) contraction of the abdominal aorta was affected neither by genotype nor by HFD. In thoracic aortae, the knockout of EGFR in EC enhanced and knockout in VSMC reduced the effect of U46619 (Supplementary Fig. SF07).

**Figure 3:**
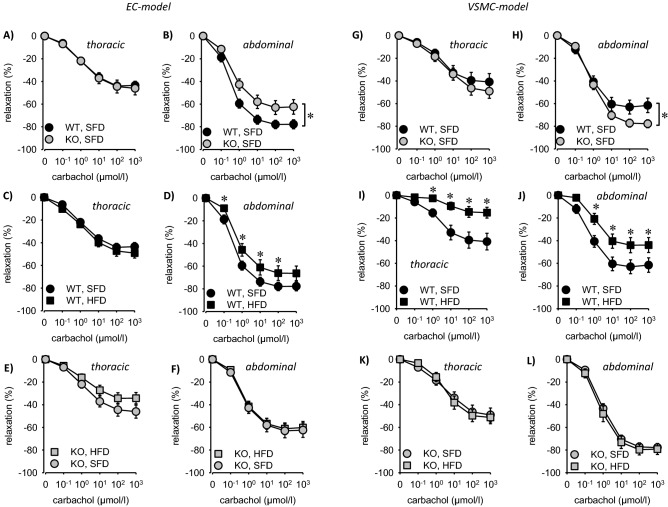
(**A**,**B**) Isometric relaxation of thoracic and abdominal aortic rings of EC-EGFR-KO and EC-EGFR-KO under SFD in response to carbachol. (**C**–**F**) Effect of HFD on isometric relaxation of thoracic (**C**,**E**) and abdominal (**D**,**F**) aortic rings in response to carbachol in EC-EGFR-WT (**C**,**D**) and EC-EGFR-KO (**E**,**F**) animals. Number of animals N(WT, SFD) = 10, N(WT, HFD) = 8, N(KO, SFD) = 8, N(KO, HFD) = 11. (**G**,**H**) Isometric relaxation of thoracic and abdominal aortic rings of VSMC-EGFR-KO and VSMC-EGFR-KO under SFD in response to carbachol. (**I**–**L**) Effect of HFD on isometric relaxation of thoracic (**I**,**K**) and abdominal (**J**,**L**) aortic rings in response to carbachol in VSMC-EGFR-WT (**I**,**J**) and VSMC-EGFR-KO (**K**,**L**) animals. Number of animals N(WT, SFD) = 10, N(WT, HFD) = 8, N(KO, SFD) = 10, N(KO, HFD) = 10. *p < 0.05 versus respective control. *SFD* standard diet, *HFD* high fat diet, *WT* wildtype, *KO* knockout, *EC* endothelial cell, *VSMC* vascular smooth muscle cell.

**Figure 4:**
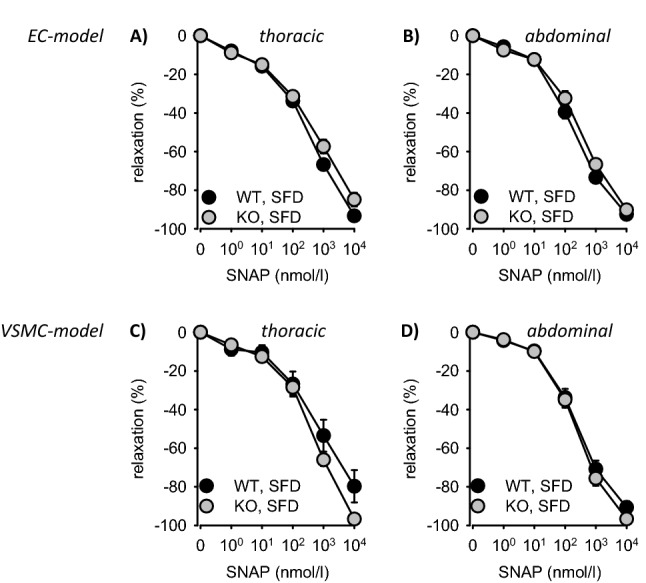
(**A**,**B**) Isometric relaxation of thoracic and abdominal aortic rings of EC-EGFR-KO and EC-EGFR-KO under SFD in response to SNAP. (**C**,**D**) Isometric relaxation of thoracic and abdominal aortic rings of VSMC-EGFR-KO and VSMC-EGFR-KO under SFD in response to SNAP. Number of animals in the EC-model: N(WT, SFD) = 10, N(KO, SFD) = 8. Number of animals in the VSMC-model: N(WT, SFD) = 10, N(KO, SFD) = 10. *p < 0.05 versus respective control. *SFD* standard diet, *HFD* high fat diet, *WT* wiltype, *KO* knockout, *EC* endothelial cell, *VSMC* vascular smooth muscle cell.

Endothelium-dependent relaxation in response to carbachol was slightly reduced in abdominal but not thoracic aortae from EC-EGFR-KO (Fig. 3). VSMC-EGFR-KO also led to a slight yet opposite effect in abdominal aorta. The impact of the NO-donor SNAP was not affected by genotype (Fig. 3). Surprisingly, aortae from EC-EGFR-WT animals responded to HFD with a mild endothelial dysfunction only in the abdominal aorta, an effect that was absent in abdominal aortae from EC-EGFR-KO animals, probably due to the preexisting mild endothelial dysfunction (Fig. 4A–D). The data from thoracic aortae indicate a trend for enhanced HFD-susceptibility in EC-EGFR-KO animals (Fig. 4). HFD reduced the action of carbachol in abdominal and thoracic aortae from WT but not from KO animals of the VSMC-EGFR model (Fig. 4E–H), as also reported previously^5^. SNAP-induced relaxation was not affected (Supplementary Figs. SF08 and SF09).

**Figure 5:**
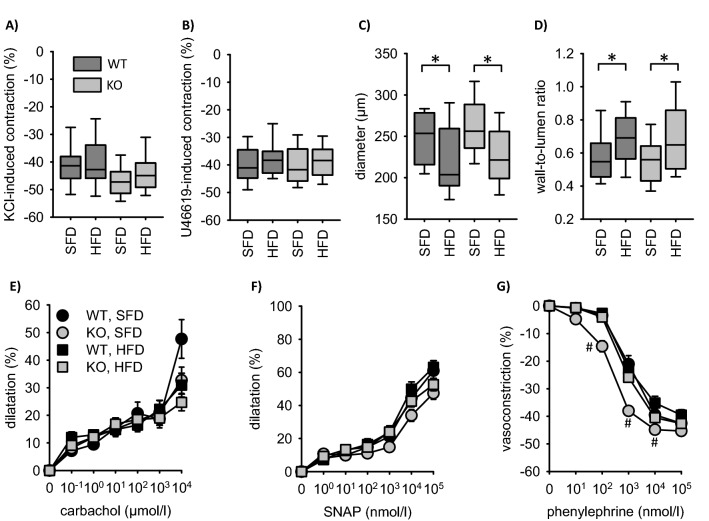
Pressure myography in mesenteric arteries of the EC-EGFR model. (**A**) Contraction in reponse to 20 mmol/l KCl. (**B**) Contraction in response to 100 nmol/l U46619. (**C**) Diameter of mesenteric arteries. (**D**) Wall-to-lumen ratio of mesenteric arteries. (**E**) Carbachol-induced dilatation. (**F**) SNAP-induced dilatation. (**G**) Phenylephrine-induced contraction. Number of animals N(WT, SFD) = 8, N(WT, HFD) = 10, N(KO, SFD) = 10, N(KO, HFD) = 14. *p < 0.05. *SFD* standard diet, *HFD* high fat diet, *WT* wildtype, *KO* knockout. *p < 0.05. ^#^p < 0.05 versus WT, SFD.

Pressure myography in mesenteric arteries (vessels with myogenic tone) showed reduced vessel diameter and increased wall-to-lumen ratio (inward remodeling) induced by HFD independent of the EC-genotype (Fig. 5). Carbachol induced relaxation was less pronounced in the EC-EGFR model (Fig. 5) compared to the VSMC-EGFR model under SFD (see Fig. 2 in^5^), indicating reduced endothelial function. EC-EGFR-KO or HFD induced no further reduction of endothelial function. The effect of SNAP was not significantly different between the various groups (Fig. 5; Fig. 2 in^5^).

As reported before, neither EGFR knockout nor HFD altered the effect of phenylephrine-induced contraction of mesenteric arteries in the VSMC-EGFR model (see Fig. 2 in^5^). In contrast, EGFR knockout enhanced phenylephrine-sensitivity in the EC-EGFR model (Fig. 5). HFD reduced the sensitivity to phenylephrine in mesenteric arteries from EC-EGFR-KO but not from EC-EGFR-WT animals.

### Aortic gene expression analysis

The effect of VSMC-EGFR-KO on aortic transcriptome has been reported before^5^. EC-EGFR-KO induced no differential expression of mRNAs under SFD (Supplementary Table ST01, Supplementary Fig. SF10) in aortas. In EC-EGFR-WT animals HFD induced only mild transcriptome alterations (3 genes were affected according to the results of differential expression analysis, with FDR 0.05, Fold Change > 1.5, FPM > 5) (Supplementary Fig. SF10). Of those three genes only Cyp2e1 was differentially expressed in EC-EGFR-KO animals treated with HFD. These data, together with the functional results above, strongly suggest that EC-EGFR is not of major importance for basal vascular function or structure—in contrast to VMSC-EGFR^2,4,5,15^.

### Renal parameters

**Figure 6:**
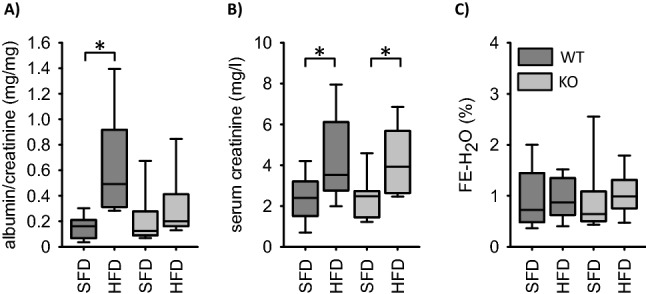
Impact of HFD-diet on functional renal parameters in the EC-EGFR-model. (**A**) Urinary albumin excretion. (**B**) Serum creatinine concentrations. (**C**) Fractional water excretion (FE). Number of animals: N = 10 for each experimental group. *p < 0.05. *SFD* standard diet, *HFD* high fat diet, *WT* wildtype, *KO* knockout.

Knockout of EGFR in EC did not affect the parameters investigated under SFD (Fig. 6). In EC-EGFR-WT animals HFD led to increased serum creatinine levels and albuminuria, similar to the effects reported previously for VSMC-EGFR-WT^5^. Knockout of EC-EGFR had no impact on the HFD induced elevated serum creatinine but attenuated albuminuria (Fig. 6). Fractional water excretion was not affected by HFD or EC-EGFR KO. The HFD-induced increase in renal weight in KO animals was accompanied by moderate histological changes, including glomerular sclerosis in both genotypes (Supplementary Fig. SF11). Proximal tubule hypertrophy or an increase in glomerular size were not observed (Supplementary Fig. SF11).

### Renal gene expression analysis

In EC-EGFR-KO, no mRNA was expressed differently under SFD. HFD induced differential expression of 310 genes in WT and 284 genes in KO animals with an overlap of 222 mRNAs and more up- than downregulated mRNAs (Supplementary Table ST02 and Supplementary Fig. SF12A). The results of Gene Ontology Term (GO) enrichment analysis, i.e. altered lipid metabolism, was not affected by EC-EGFR knockout (Supplementary Table ST03) in contrast to the previously reported effect of VSMC-EGFR-KO^5^. Furthermore, upstream regulator analysis by IPA® also provided no evidence for a different impact of HFD on EC-EGFR-KO as compared to EC-EGFR-WT animals (Supplementary Fig. SF12B).

## Discussion

From our results, we conclude that EC-EGFR is of minor importance for basal physiological renovascular function or HFD-induced alterations compared to VSMC-EGFR. Gross organ morphology, aortic and renal transcriptome, mesenteric artery or renal function were not affected substantially by EC-EGFR-KO under SFD. We detected a slight endothelial dysfunction in the abdominal aorta from EC-EGFR-KO animals under SFD that was not observed in thoracic aorta or mesenteric arteries, indicating limited systemic impact of the KO. This conclusion is supported by the fact that blood pressure did not differ between EC-EGFR-KO and WT animals. Of course, we do not exclude the possibility of a local regulatory importance of EC-EGFR, for example during metabolic blood flow control. Regarding the interpretation of results obtained with EC-KO models the problem of non-EC expression in pan-EC-KO models, as reviewed by Payne et al.^28^ has to be considered. Non-EC-expression in haematopoietic cells has been reported for the pan-EC models Tie2, VE-cadherin and Flk1. In cases when substantial KO effects are observed a possible contribution of haematopoietic cells must be discussed. Because EGFR-KO in our model exerted no major effect, the conclusions that EGFR-KO is of minor importance is still valid in our opinion. Furthermore, it has to be considered that we used the non-inducible Tie2-CRE model in this initial study, in order to investigate whether major effects of EC-EGFR-KO can be observed, whereas for VSMC-EGFR we previously had used a non-inducible and an inducible CRE-system^2,4,5,15^ with similar results for the basal effects. In case of major effects in the Tie2-CRE model confirmation in an inducible system (e.g. the tamoxifen-inducible VE-cadherin-CRE model^28^) should be performed. However, so far the effects observed are only mild in the Tie2-CRE-model. Nevertheless, we started with the implementation of the inducible VE-cadherin-KO model for EC-EGFR-KO. Unfortunately, the knock-down efficiency was very variable in our first cohorts. We will try to improve the protocol for EC-EGFR-KO so that this model can eventually be used to study EC-EGFR further in future studies.

Possibly, VSMC-KO and EC-KO play opposite roles in abdominal aorta under basal conditions, because VSMC-EGFR-KO led to enhanced endothelial dependent and EC-EGFR-KO to reduced relaxation (Fig. 3). As this effect of EC-EGFR-KO was not observed in thoracic aorta or mesenteric arteries from EC-EGFR-KO mice, presumably it is of minor systemic relevance (see also Supplementary Fig. SF04). Furthermore, we observed a slight increase in phenylephrine sensitivity of mesenteric arteries in EC-EGFR-KO animals, which was not observed in the aorta of EC-EGFR-KO animals or in mesenteric arteries from VSMC-EGFR-KO animals. Yet, we could not show an acute systemic impact of phenylephrine, as the acute increase in blood pressure was not affected by EC-EGFR-KO (Supplementary Fig. SF13). HFD-induced cardiovascular effects are virtually identical in EC-EGFR-WT and EC-EGFR-KO animals, with the exception of the mild endothelial dysfunction of abdominal aorta that was not observed in EC-EGFR-KO, most probably due to the already preexisting endothelial dysfunction (Fig. 3). Moreover, HFD-induced albuminuria was less pronounced in EC-EGFR-KO animals whereas the HFD-induced decrease in estimated glomerular filtration rate (from serum creatinine) was not prevented. These finding keeps the option of a partial protection of the glomerular filtration barrier without prevention of overall HFD-induced vascular remodeling, which is supported by mesenteric artery wall thickening. Altogether, these findings are in contrast to VSMC-EGFR that plays an important role under physiological and pathophysiological conditions, as reported previously^2,4,5,15^.

The limited impact of EC-EGFR on vessel wall homeostasis is also reflected by the lack of changes of the aortic mRNA-transcriptome in EC-EGFR-KO animals under SFD. Of course our data do not exclude alterations in mRNA expression restricted to endothelial cells, due to the fact that endothelial cells represent only a minority of vascular wall cell population. This question has to be addressed using isolated endothelial cells in future studies.

HFD induced virtually no changes of the aortic transcriptome in EC-EGFR-WT animals, unlike in the VSMC-EGFR model^5^. When we compared the transcriptome of aortas from WT, SFD animals of the VSMC-EGFR model with the EC-EGFR model, we observed distinct differences with respect to the extracellular matrix, indicating a more “fibrotic” vascular type already under control conditions (Supplementary Figs. SF14 and S15). This fits to suggested functional consequences of the slightly different genetic background we had to use for the two models^27,29–31^, because B6.Cg-Tg(Tek-cre)1Ywa animals are of C57BL/6J background and B6.FVB-Tg(Myh11-cre/ERT2)1Soff of C57BL/6N background (https://www.jax.org/strain/019079, section Details, Development). It has been reported that this difference can lead to subtle yet relevant phenotypic differences, comprising higher blood glucose concentrations, glucose intolerance, lower insulin secretion and lower oxygen consumption in C57BL/6 J, possibly due to the *Nnt* mutation^27^. This is of course a limitation of our study with respect to HFD, because a comprised glucose tolerance in WT animals might disguise obesity-induced alterations, although our data show that the animals developed obesity and hyperglycemia.

In contrast to the vascular phenotype, HFD-induced renal alterations were comparable in EC-EGFR-WT and VSMC-EGFR-WT animals, reflecting the metabolic syndrome (reduced estimated glomerular filtration, albuminuria, glomerulosclerosis and a shift in the transcriptome in response to the enhanced lipid load). However, EC-EGFR-KO was almost without effect on HFD-induced alterations, in contrast to VSMC-EGFR-KO, as reported before^5^. The less pronounced albuminuria in EC-EGFR-KO animals suggests a certain protective effect but cannot be explained or classified at the moment. Because the impairment of estimated glomerular filtration as well as glomerular sclerosis was not prevented, we assume that there is not major protective effect.

The scattered analysis of organ weights in a larger population indicates that, even under standardized laboratory conditions, there are subgroups of more susceptible animals, especially in the VSMC-EGFR model. This was evident for the effect of HFD on renal weight in VSMC-EGFR-KO for the effect of EGFR-KO in VSMC on lung weight already under SFD. Assuming that the enhanced lung weight results from congestion, these data suggest a yet unrecognized importance of VSMC-EGFR for the cardiopulmonary system in a subgroup of animals. The underlying mechanisms for the difference in susceptibility as well as for the pathological lung phenotype are not known and should be investigated in more depth in future studies in order to identify potential genetic risk variants.

## Conclusions

In summary, our results show that EC-EGFR, in comparison to VSMC-EGFR, is of minor importance for basal vascular and renal function, as well as for HFD-induced functional vascular remodeling, endothelial dysfunction and renal end-organ damage. Thus, therapeutic targeting strategies aiming at vascular EGFR should put their conceptual focus on VSMC because major EC-derived effects are unlikely.

## Supplementary Information

Supplementary Information 1.

Supplementary Information 2.

Supplementary Information 3.

Supplementary Information 4.

## Data Availability

Datasets generated during and/or analysed during the current study are available in the gene expression omnibus database with the study identity GSE144838 and GSE158197. (https://www.ncbi.nlm.nih.gov/geo/query/acc.cgi?acc=GSE144838) (https://www.ncbi.nlm.nih.gov/geo/query/acc.cgi?acc=GSE158197). All further data generated or analysed during this study are included in this published article and its supplementary information files.

## Abbreviations

AII: Angiotensin II
DMT2: Diabetes mellitus type 2
EC: Endothelial cell
EGFR: EGF receptor
ErbB1: EGF receptor
FDR: False discovery rate
FPM: Fragments per million
GO: Gene ontology
HFD: High-fat diet
IPA: Ingenuity Pathway Analysis
KO: Knock out
PAS: Periodic acid–Schiff’s reagent
SFD: Standard- fat diet
SNAP: S-nitroso-*N*-acetyl-DL-penicillamine
TL: Tibia length
VSM: Vascular smooth muscle
VSMC: Vascular smooth muscle cell
WT: Wild-type
