# Supplementary material for: Endothelial epidermal growth factor receptor is of minor importance for vascular and renal function and obesity-induced dysfunction in mice
Source: Sci Rep. 2021 Mar 31;11:7269. doi: 10.1038/s41598-021-86587-3 (PMC8012653; doi:10.1038/s41598-021-86587-3)
Supplement: Supplementary file 1 — Supplementary Information 1. [file 41598_2021_86587_MOESM1_ESM.pptx]

## Slide 1
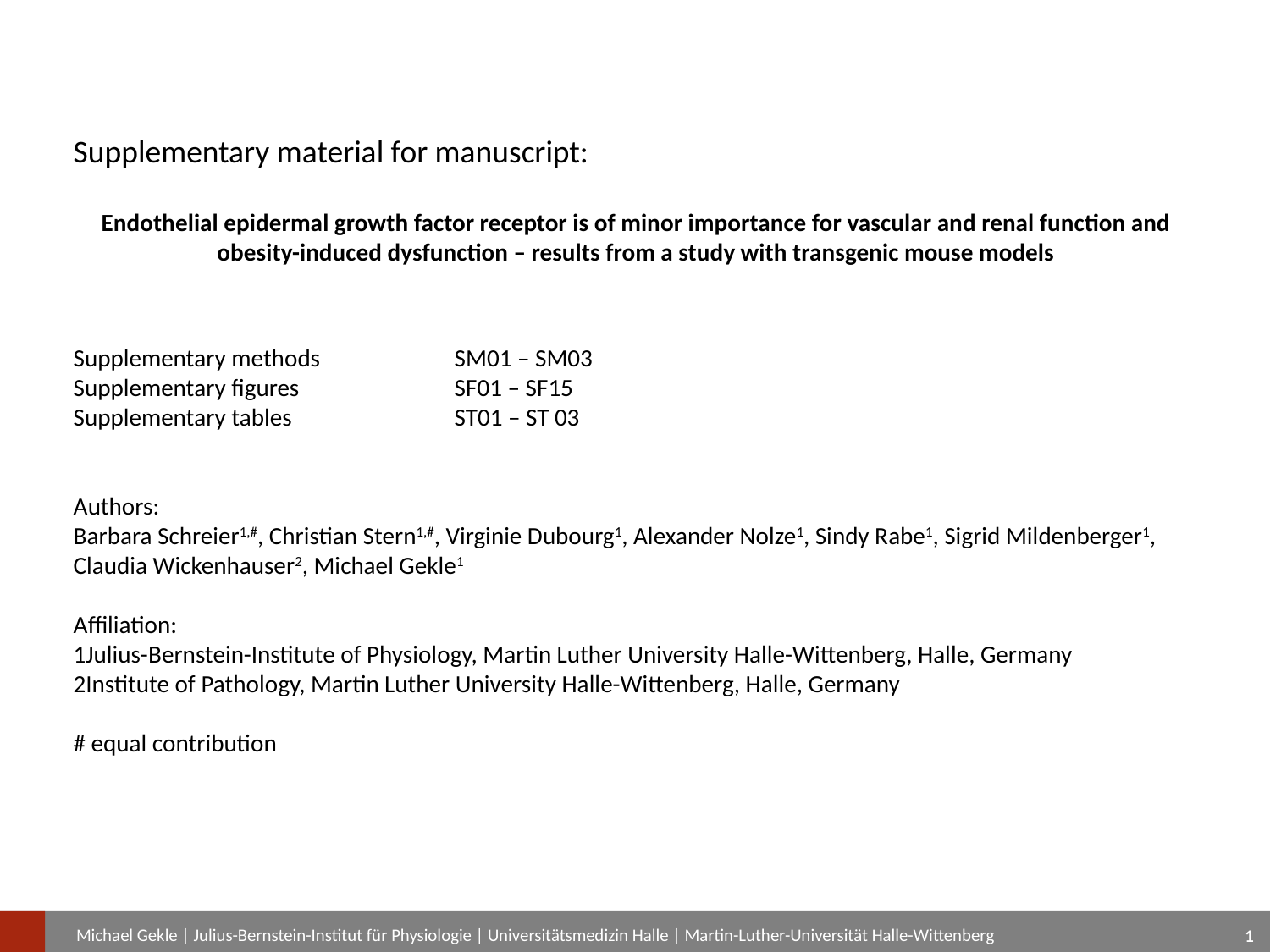

Supplementary material for manuscript:
Endothelial epidermal growth factor receptor is of minor importance for vascular and renal function and obesity-induced dysfunction – results from a study with transgenic mouse models
Supplementary methods		SM01 – SM03
Supplementary figures		SF01 – SF15
Supplementary tables		ST01 – ST 03
Authors:
Barbara Schreier1,#, Christian Stern1,#, Virginie Dubourg1, Alexander Nolze1, Sindy Rabe1, Sigrid Mildenberger1, Claudia Wickenhauser2, Michael Gekle1
Affiliation:
1Julius-Bernstein-Institute of Physiology, Martin Luther University Halle-Wittenberg, Halle, Germany
2Institute of Pathology, Martin Luther University Halle-Wittenberg, Halle, Germany
# equal contribution
1

## Slide 2
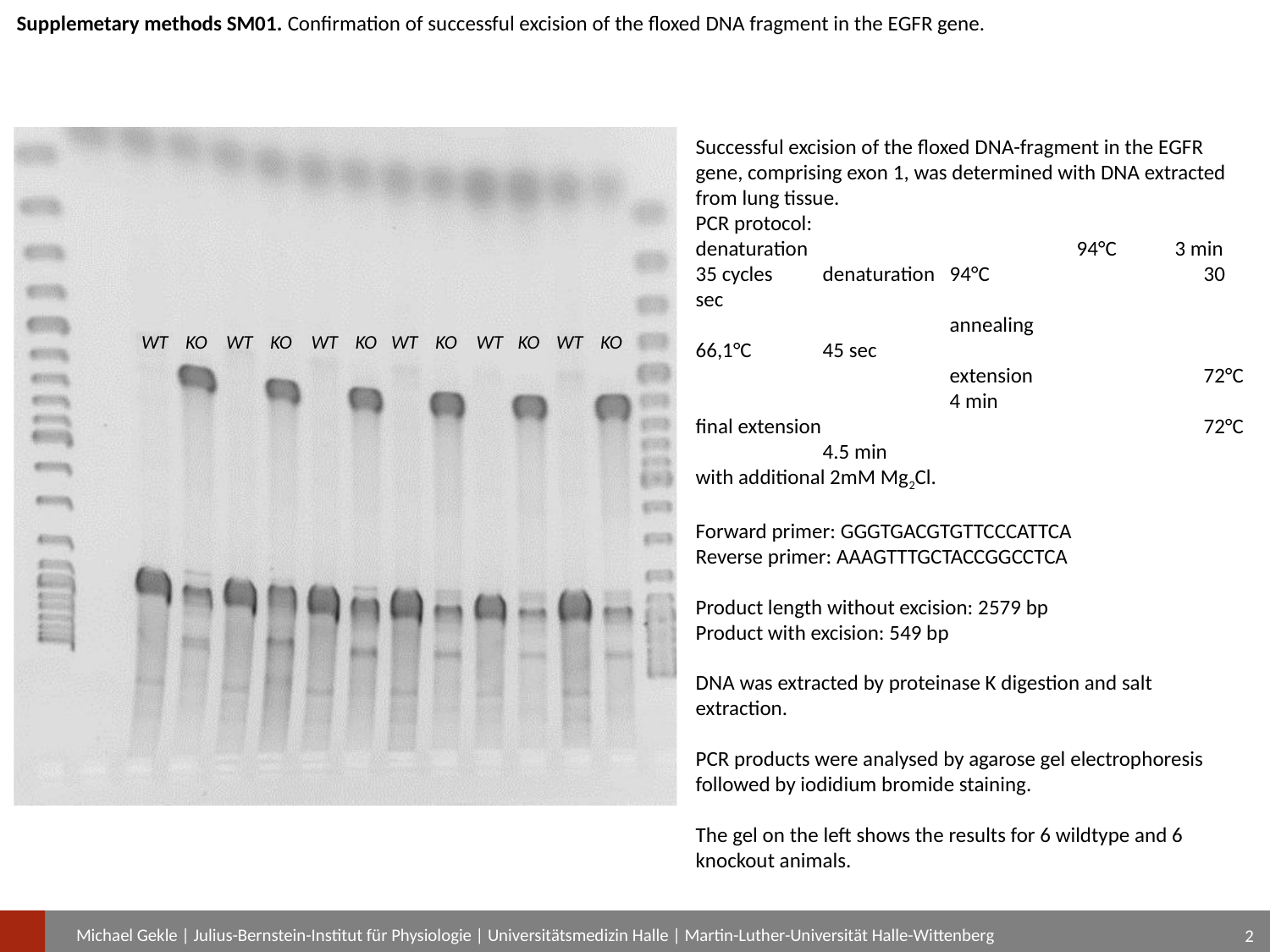

Supplemetary methods SM01. Confirmation of successful excision of the floxed DNA fragment in the EGFR gene.
WT
KO
WT
KO
WT
KO
WT
KO
WT
KO
WT
KO
Successful excision of the floxed DNA-fragment in the EGFR gene, comprising exon 1, was determined with DNA extracted from lung tissue.
PCR protocol:
denaturation			94°C 3 min
35 cycles	denaturation	94°C		30 sec
	 	annealing 		66,1°C	45 sec
	 	extension		72°C		4 min
final extension 			72°C	4.5 min
with additional 2mM Mg2Cl.
Forward primer: GGGTGACGTGTTCCCATTCA
Reverse primer: AAAGTTTGCTACCGGCCTCA
Product length without excision: 2579 bp
Product with excision: 549 bp
DNA was extracted by proteinase K digestion and salt extraction.
PCR products were analysed by agarose gel electrophoresis followed by iodidium bromide staining.
The gel on the left shows the results for 6 wildtype and 6 knockout animals.
2

## Slide 3
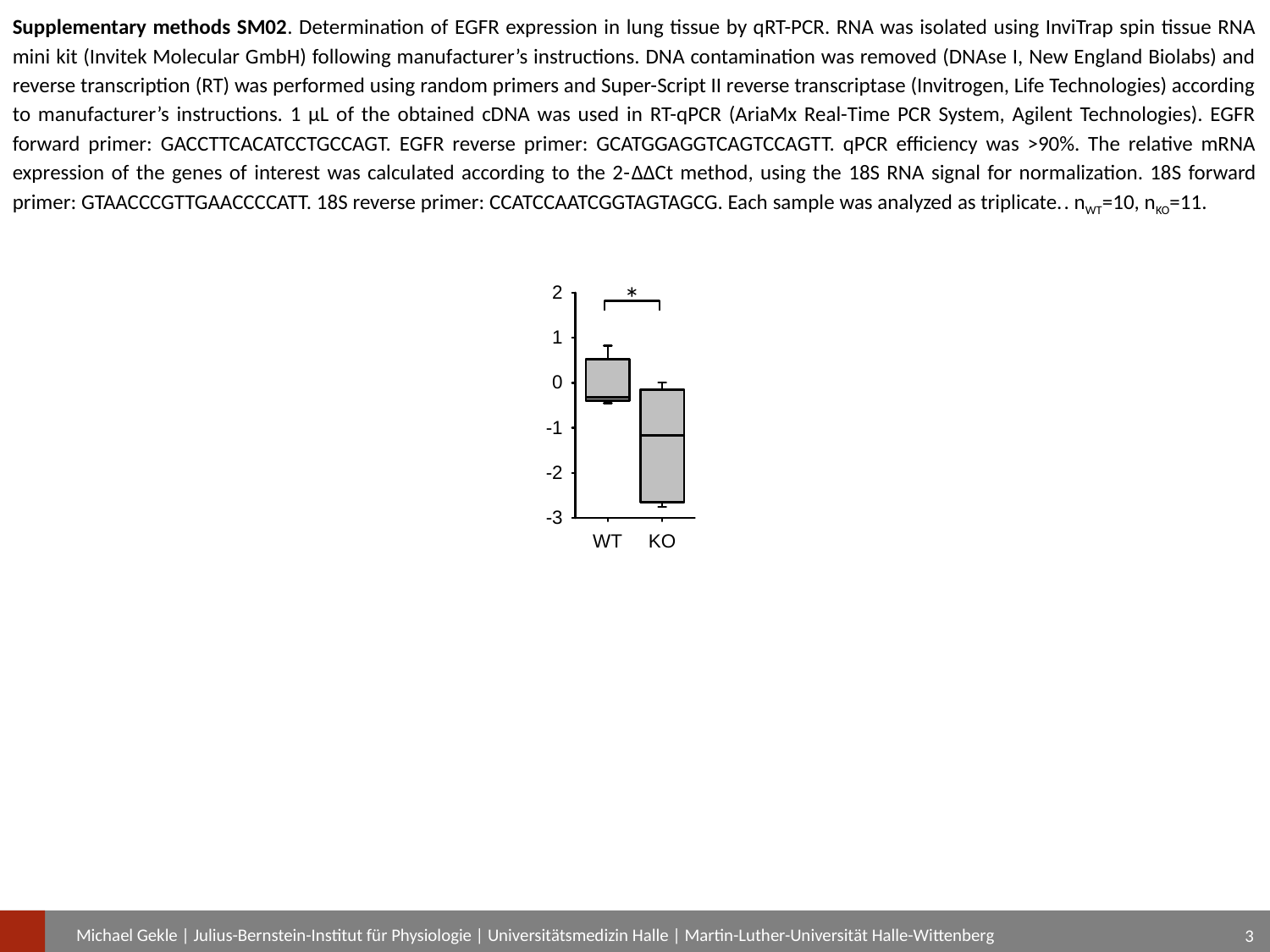

Supplementary methods SM02. Determination of EGFR expression in lung tissue by qRT-PCR. RNA was isolated using InviTrap spin tissue RNA mini kit (Invitek Molecular GmbH) following manufacturer’s instructions. DNA contamination was removed (DNAse I, New England Biolabs) and reverse transcription (RT) was performed using random primers and Super-Script II reverse transcriptase (Invitrogen, Life Technologies) according to manufacturer’s instructions. 1 µL of the obtained cDNA was used in RT-qPCR (AriaMx Real-Time PCR System, Agilent Technologies). EGFR forward primer: GACCTTCACATCCTGCCAGT. EGFR reverse primer: GCATGGAGGTCAGTCCAGTT. qPCR efficiency was >90%. The relative mRNA expression of the genes of interest was calculated according to the 2-ΔΔCt method, using the 18S RNA signal for normalization. 18S forward primer: GTAACCCGTTGAACCCCATT. 18S reverse primer: CCATCCAATCGGTAGTAGCG. Each sample was analyzed as triplicate.. nWT=10, nKO=11.
*
3

## Slide 4
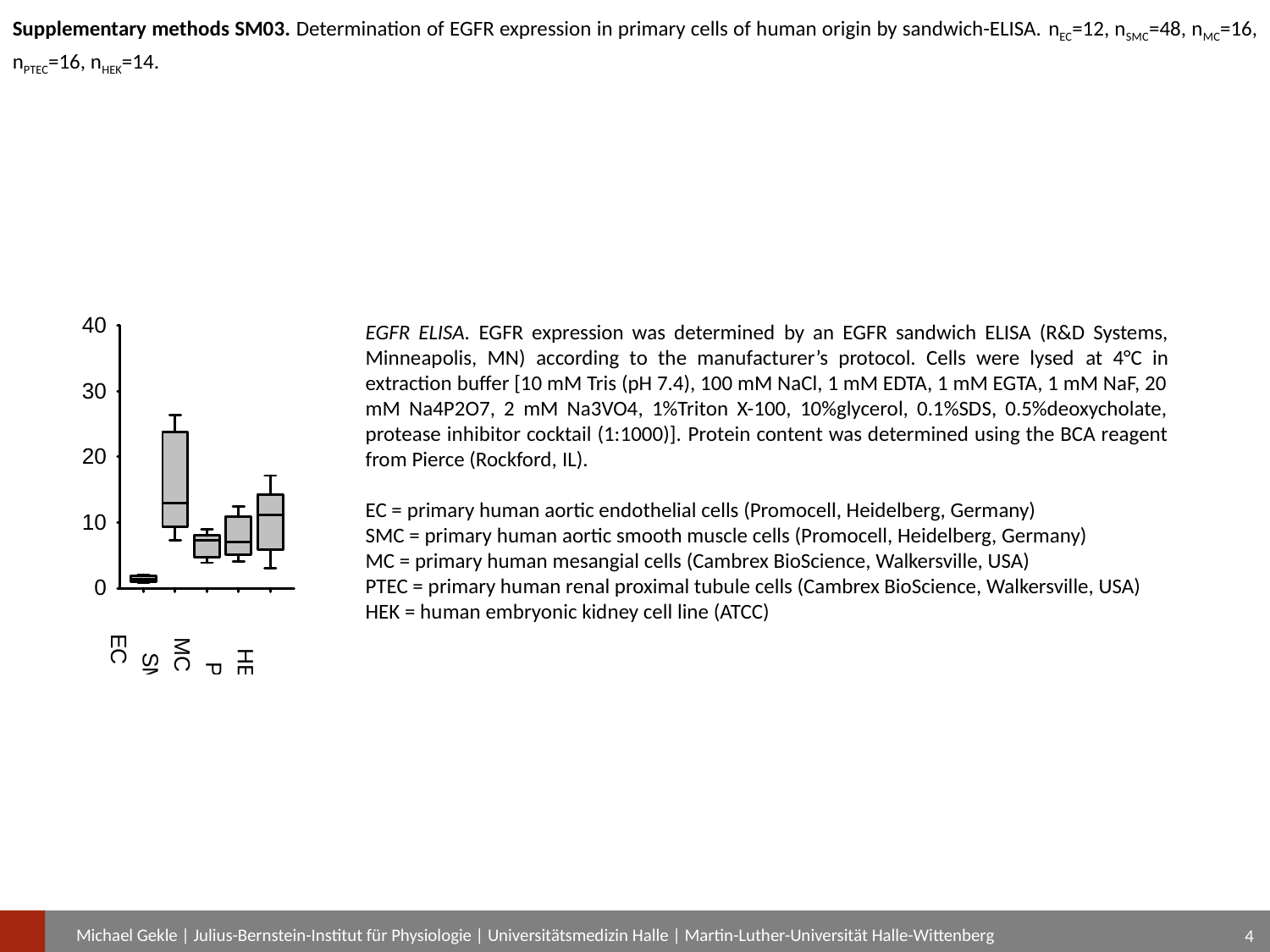

Supplementary methods SM03. Determination of EGFR expression in primary cells of human origin by sandwich-ELISA. nEC=12, nSMC=48, nMC=16, nPTEC=16, nHEK=14.
EGFR ELISA. EGFR expression was determined by an EGFR sandwich ELISA (R&D Systems, Minneapolis, MN) according to the manufacturer’s protocol. Cells were lysed at 4°C in extraction buffer [10 mM Tris (pH 7.4), 100 mM NaCl, 1 mM EDTA, 1 mM EGTA, 1 mM NaF, 20 mM Na4P2O7, 2 mM Na3VO4, 1%Triton X-100, 10%glycerol, 0.1%SDS, 0.5%deoxycholate, protease inhibitor cocktail (1:1000)]. Protein content was determined using the BCA reagent from Pierce (Rockford, IL).
EC = primary human aortic endothelial cells (Promocell, Heidelberg, Germany)
SMC = primary human aortic smooth muscle cells (Promocell, Heidelberg, Germany)
MC = primary human mesangial cells (Cambrex BioScience, Walkersville, USA)
PTEC = primary human renal proximal tubule cells (Cambrex BioScience, Walkersville, USA)
HEK = human embryonic kidney cell line (ATCC)
4

## Slide 5
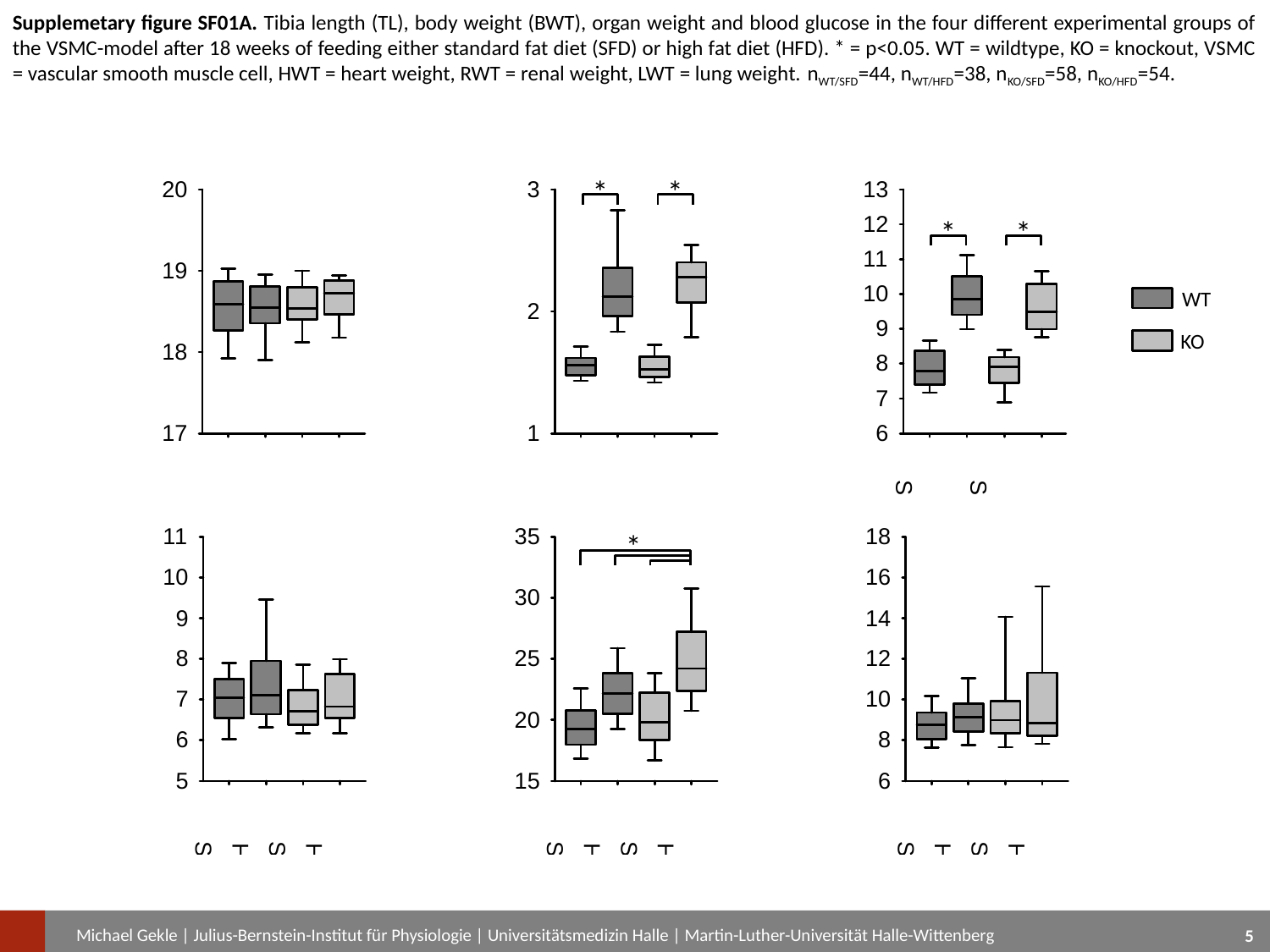

Supplemetary figure SF01A. Tibia length (TL), body weight (BWT), organ weight and blood glucose in the four different experimental groups of the VSMC-model after 18 weeks of feeding either standard fat diet (SFD) or high fat diet (HFD). * = p<0.05. WT = wildtype, KO = knockout, VSMC = vascular smooth muscle cell, HWT = heart weight, RWT = renal weight, LWT = lung weight. nWT/SFD=44, nWT/HFD=38, nKO/SFD=58, nKO/HFD=54.
*
*
*
*
WT
KO
*
5

## Slide 6
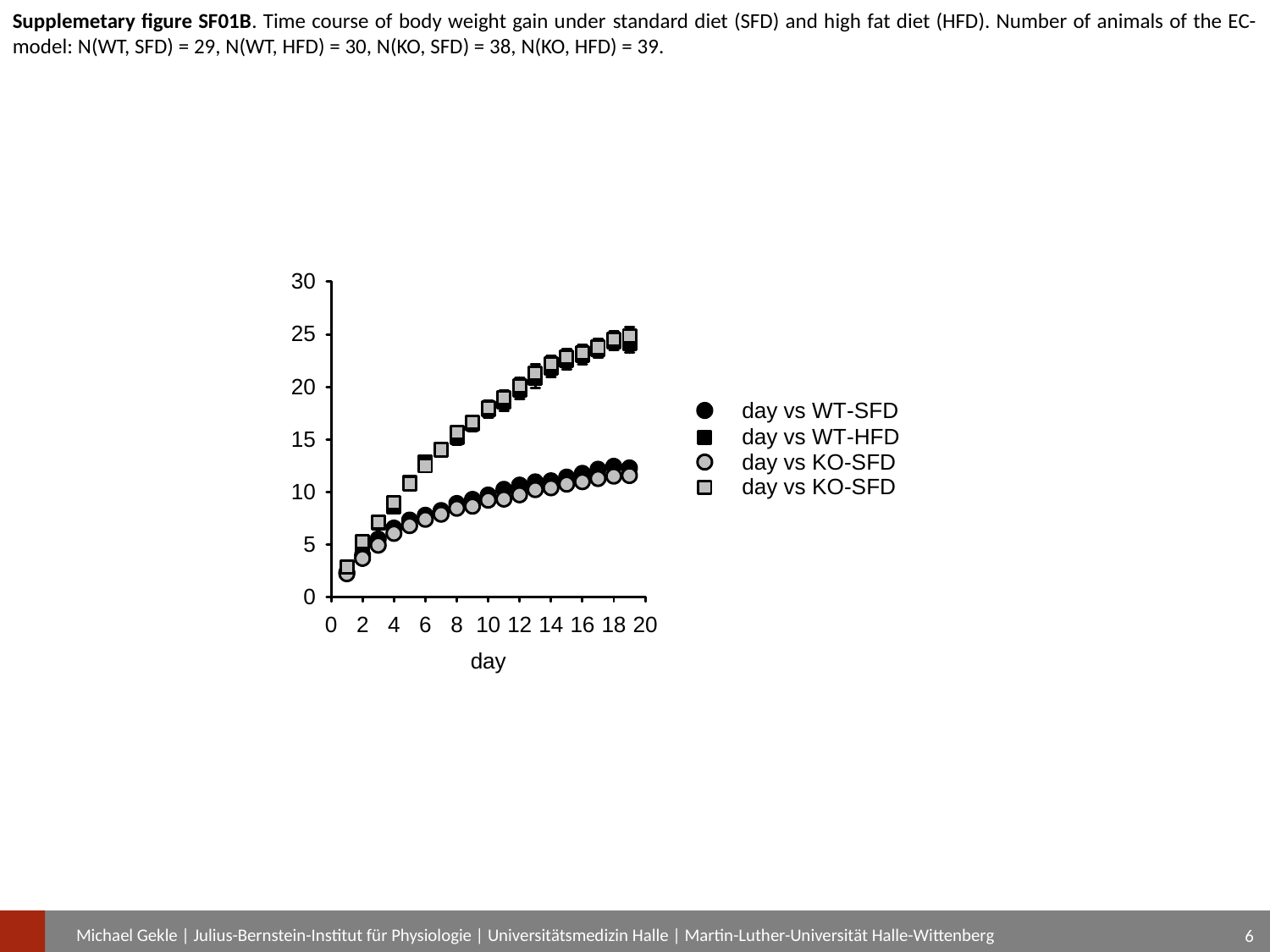

Supplemetary figure SF01B. Time course of body weight gain under standard diet (SFD) and high fat diet (HFD). Number of animals of the EC-model: N(WT, SFD) = 29, N(WT, HFD) = 30, N(KO, SFD) = 38, N(KO, HFD) = 39.
6

## Slide 7
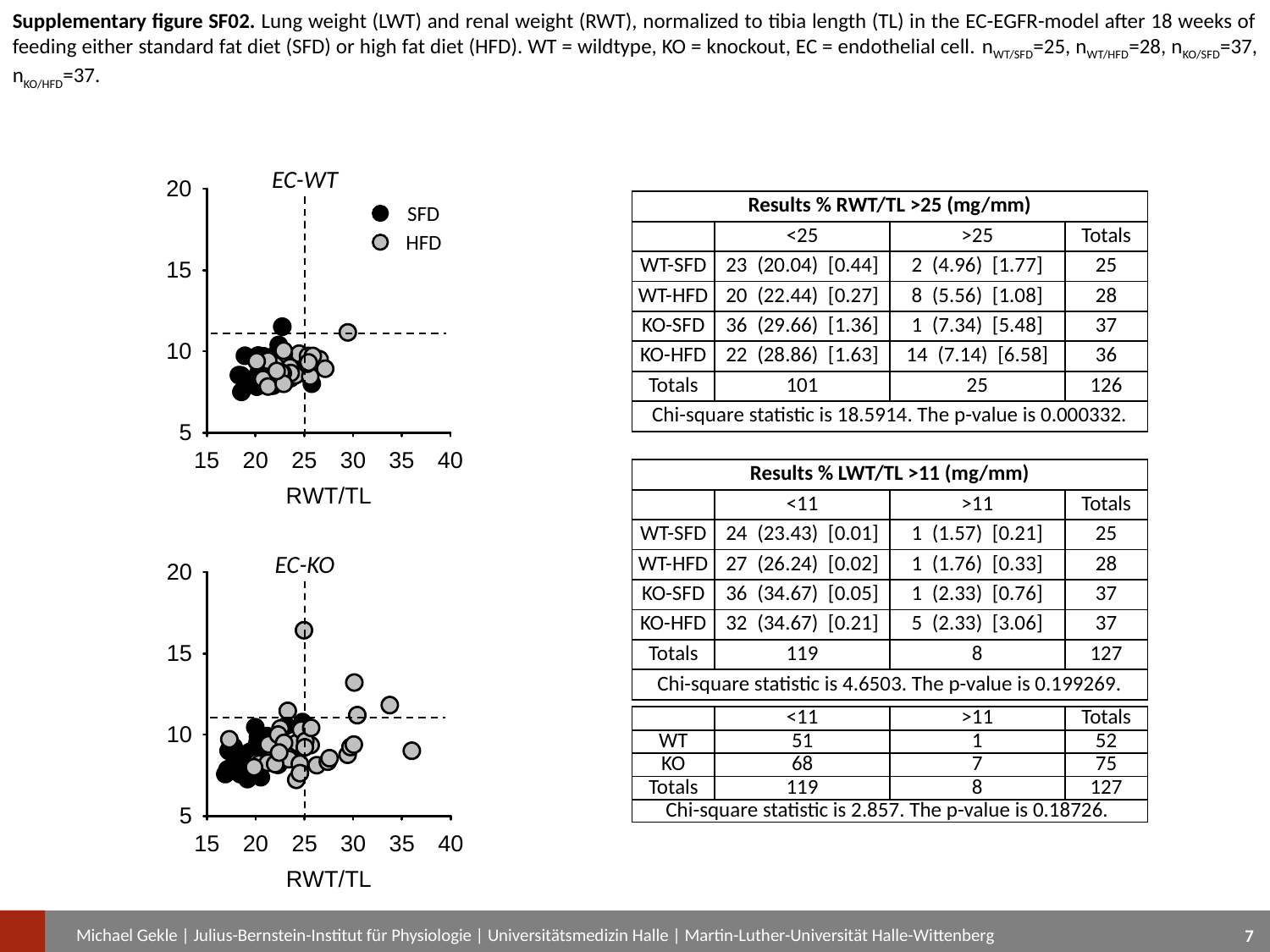

Supplementary figure SF02. Lung weight (LWT) and renal weight (RWT), normalized to tibia length (TL) in the EC-EGFR-model after 18 weeks of feeding either standard fat diet (SFD) or high fat diet (HFD). WT = wildtype, KO = knockout, EC = endothelial cell. nWT/SFD=25, nWT/HFD=28, nKO/SFD=37, nKO/HFD=37.
EC-WT
| Results % RWT/TL >25 (mg/mm) | | | |
| --- | --- | --- | --- |
| | <25 | >25 | Totals |
| WT-SFD | 23  (20.04)  [0.44] | 2  (4.96)  [1.77] | 25 |
| WT-HFD | 20  (22.44)  [0.27] | 8  (5.56)  [1.08] | 28 |
| KO-SFD | 36  (29.66)  [1.36] | 1  (7.34)  [5.48] | 37 |
| KO-HFD | 22  (28.86)  [1.63] | 14  (7.14)  [6.58] | 36 |
| Totals | 101 | 25 | 126 |
| Chi-square statistic is 18.5914. The p-value is 0.000332. | | | |
SFD
HFD
| Results % LWT/TL >11 (mg/mm) | | | |
| --- | --- | --- | --- |
| | <11 | >11 | Totals |
| WT-SFD | 24  (23.43)  [0.01] | 1  (1.57)  [0.21] | 25 |
| WT-HFD | 27  (26.24)  [0.02] | 1  (1.76)  [0.33] | 28 |
| KO-SFD | 36  (34.67)  [0.05] | 1  (2.33)  [0.76] | 37 |
| KO-HFD | 32  (34.67)  [0.21] | 5  (2.33)  [3.06] | 37 |
| Totals | 119 | 8 | 127 |
| Chi-square statistic is 4.6503. The p-value is 0.199269. | | | |
EC-KO
| | <11 | >11 | Totals |
| --- | --- | --- | --- |
| WT | 51 | 1 | 52 |
| KO | 68 | 7 | 75 |
| Totals | 119 | 8 | 127 |
| Chi-square statistic is 2.857. The p-value is 0.18726. | | | |
7

## Slide 8
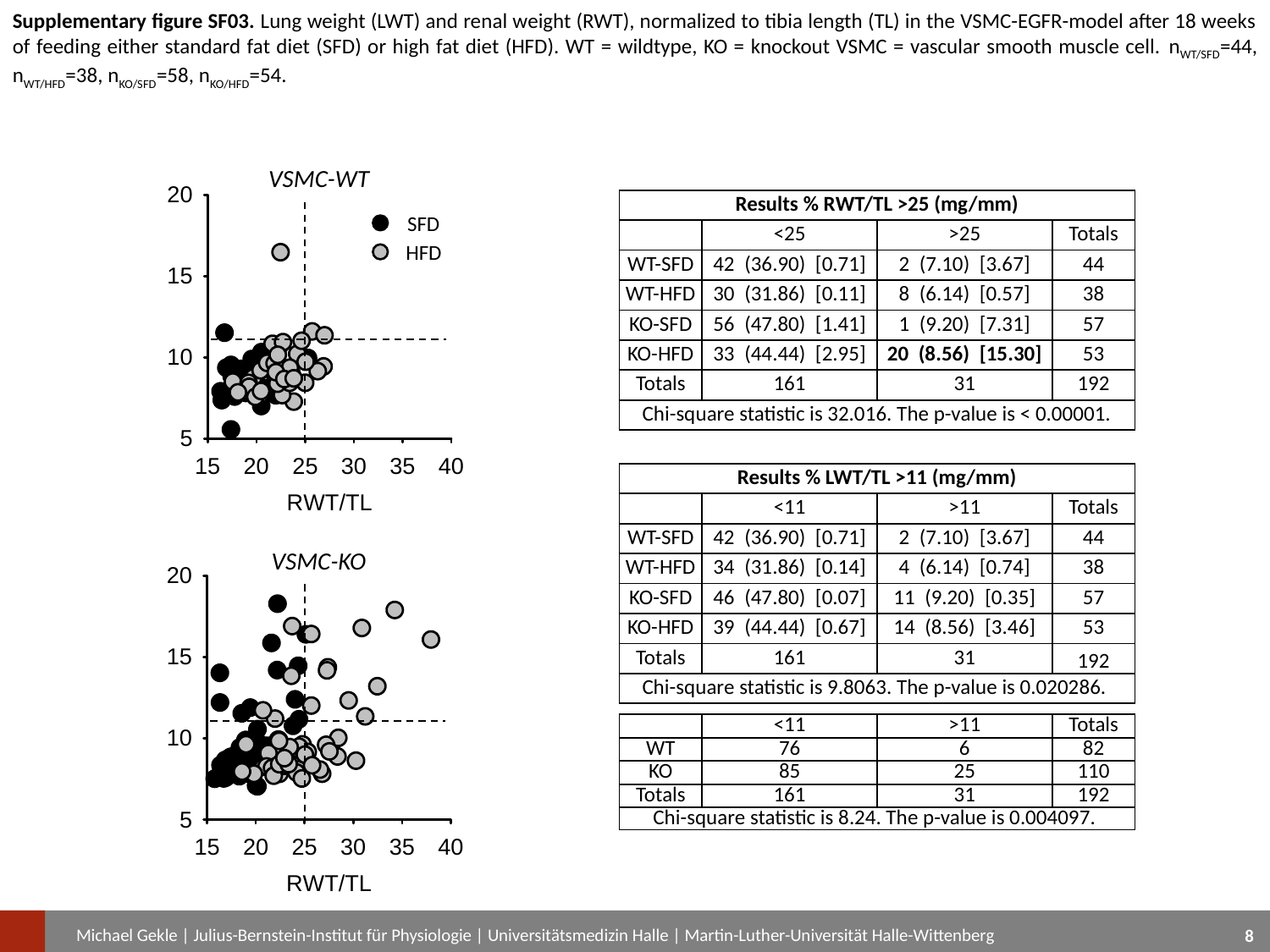

Supplementary figure SF03. Lung weight (LWT) and renal weight (RWT), normalized to tibia length (TL) in the VSMC-EGFR-model after 18 weeks of feeding either standard fat diet (SFD) or high fat diet (HFD). WT = wildtype, KO = knockout VSMC = vascular smooth muscle cell. nWT/SFD=44, nWT/HFD=38, nKO/SFD=58, nKO/HFD=54.
VSMC-WT
| Results % RWT/TL >25 (mg/mm) | | | |
| --- | --- | --- | --- |
| | <25 | >25 | Totals |
| WT-SFD | 42  (36.90)  [0.71] | 2  (7.10)  [3.67] | 44 |
| WT-HFD | 30  (31.86)  [0.11] | 8  (6.14)  [0.57] | 38 |
| KO-SFD | 56  (47.80)  [1.41] | 1  (9.20)  [7.31] | 57 |
| KO-HFD | 33  (44.44)  [2.95] | 20  (8.56)  [15.30] | 53 |
| Totals | 161 | 31 | 192 |
| Chi-square statistic is 32.016. The p-value is < 0.00001. | | | |
SFD
HFD
| Results % LWT/TL >11 (mg/mm) | | | |
| --- | --- | --- | --- |
| | <11 | >11 | Totals |
| WT-SFD | 42  (36.90)  [0.71] | 2  (7.10)  [3.67] | 44 |
| WT-HFD | 34  (31.86)  [0.14] | 4  (6.14)  [0.74] | 38 |
| KO-SFD | 46  (47.80)  [0.07] | 11  (9.20)  [0.35] | 57 |
| KO-HFD | 39  (44.44)  [0.67] | 14  (8.56)  [3.46] | 53 |
| Totals | 161 | 31 | 192 |
| Chi-square statistic is 9.8063. The p-value is 0.020286. | | | |
VSMC-KO
| | <11 | >11 | Totals |
| --- | --- | --- | --- |
| WT | 76 | 6 | 82 |
| KO | 85 | 25 | 110 |
| Totals | 161 | 31 | 192 |
| Chi-square statistic is 8.24. The p-value is 0.004097. | | | |
8

## Slide 9
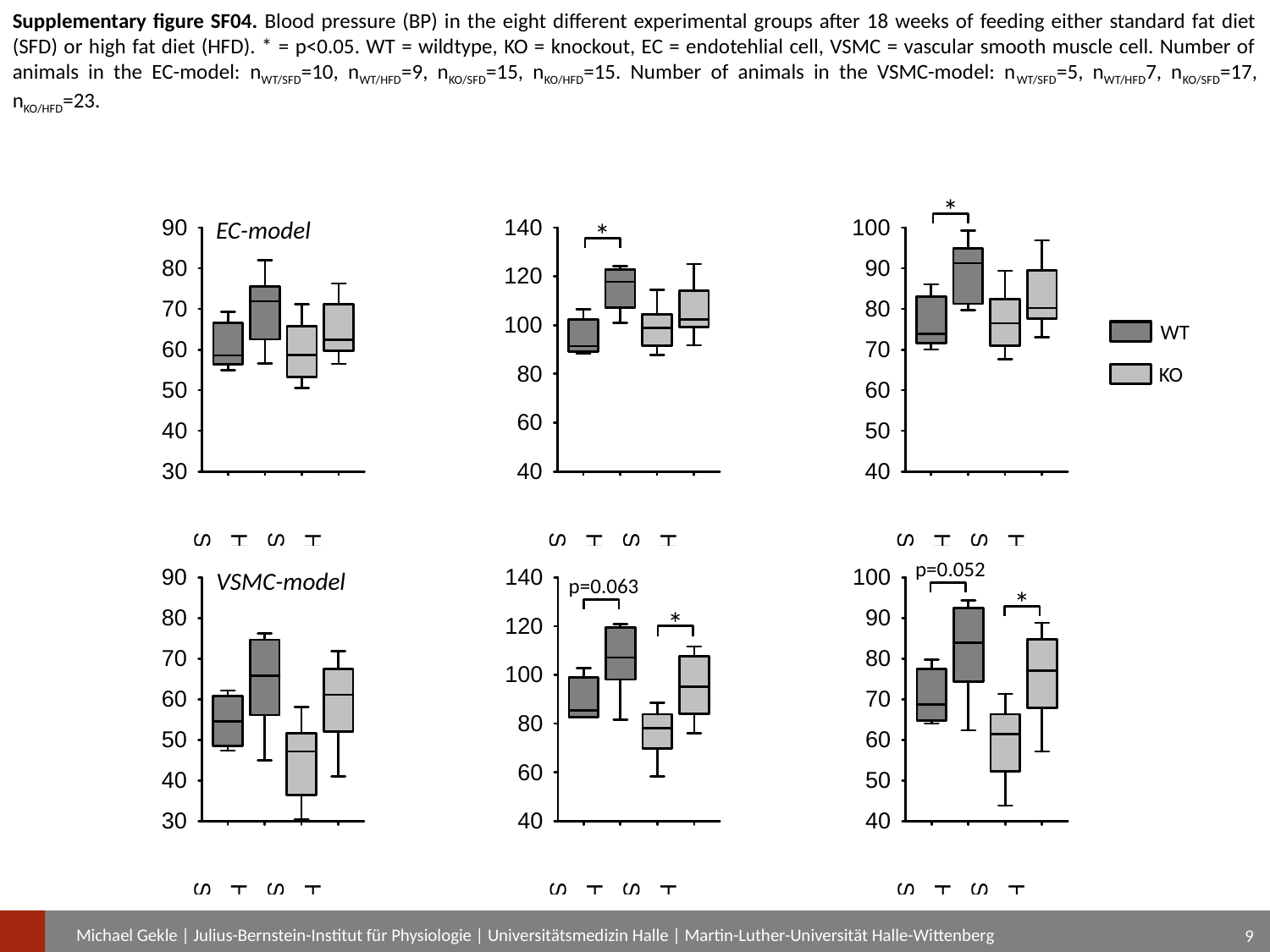

Supplementary figure SF04. Blood pressure (BP) in the eight different experimental groups after 18 weeks of feeding either standard fat diet (SFD) or high fat diet (HFD). * = p<0.05. WT = wildtype, KO = knockout, EC = endotehlial cell, VSMC = vascular smooth muscle cell. Number of animals in the EC-model: nWT/SFD=10, nWT/HFD=9, nKO/SFD=15, nKO/HFD=15. Number of animals in the VSMC-model: nWT/SFD=5, nWT/HFD7, nKO/SFD=17, nKO/HFD=23.
*
EC-model
*
WT
KO
p=0.052
VSMC-model
p=0.063
*
*
9

## Slide 10
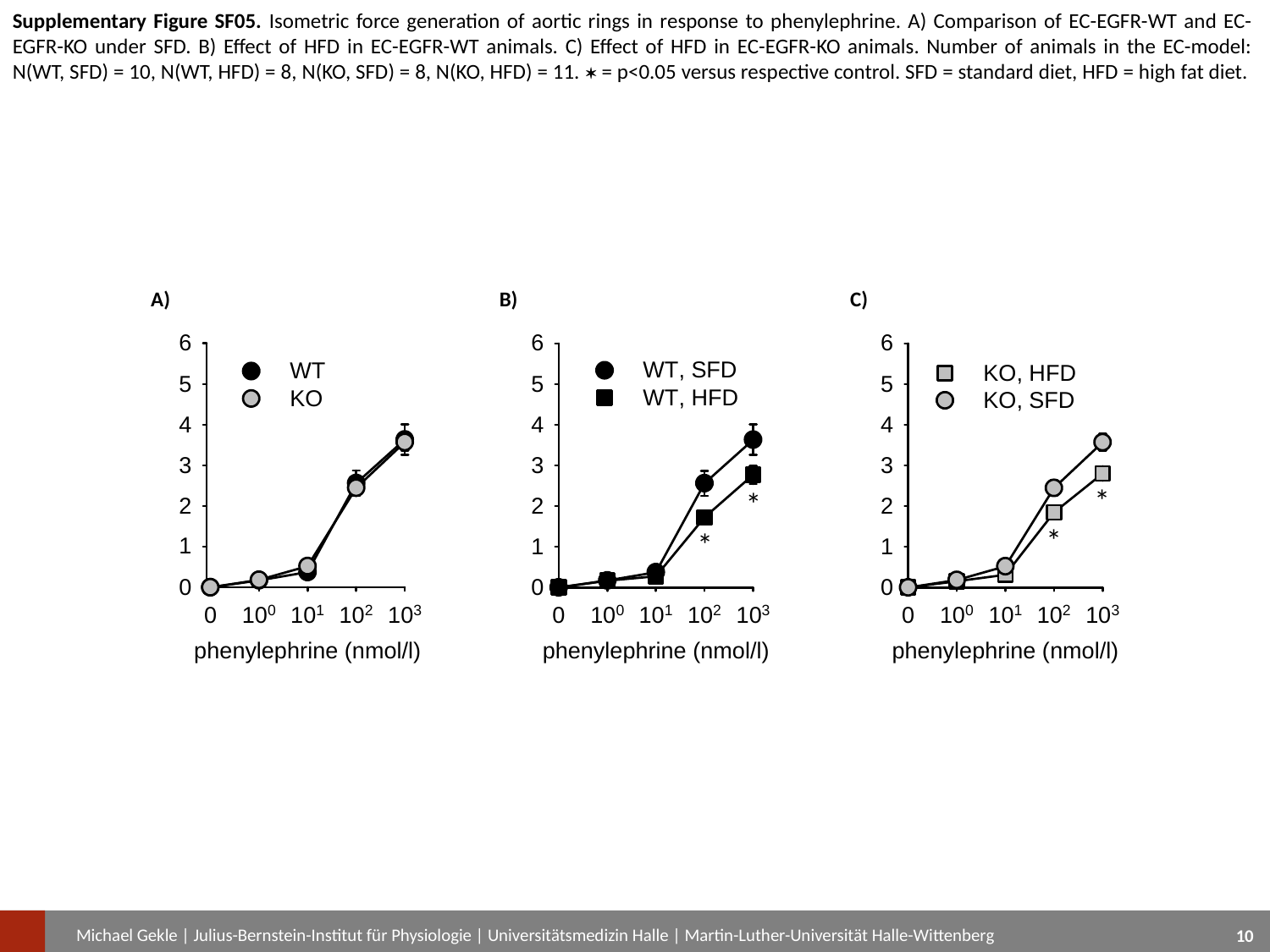

Supplementary Figure SF05. Isometric force generation of aortic rings in response to phenylephrine. A) Comparison of EC-EGFR-WT and EC-EGFR-KO under SFD. B) Effect of HFD in EC-EGFR-WT animals. C) Effect of HFD in EC-EGFR-KO animals. Number of animals in the EC-model: N(WT, SFD) = 10, N(WT, HFD) = 8, N(KO, SFD) = 8, N(KO, HFD) = 11.  = p<0.05 versus respective control. SFD = standard diet, HFD = high fat diet.
A)
B)
C)
*
*
*
*
10

## Slide 11
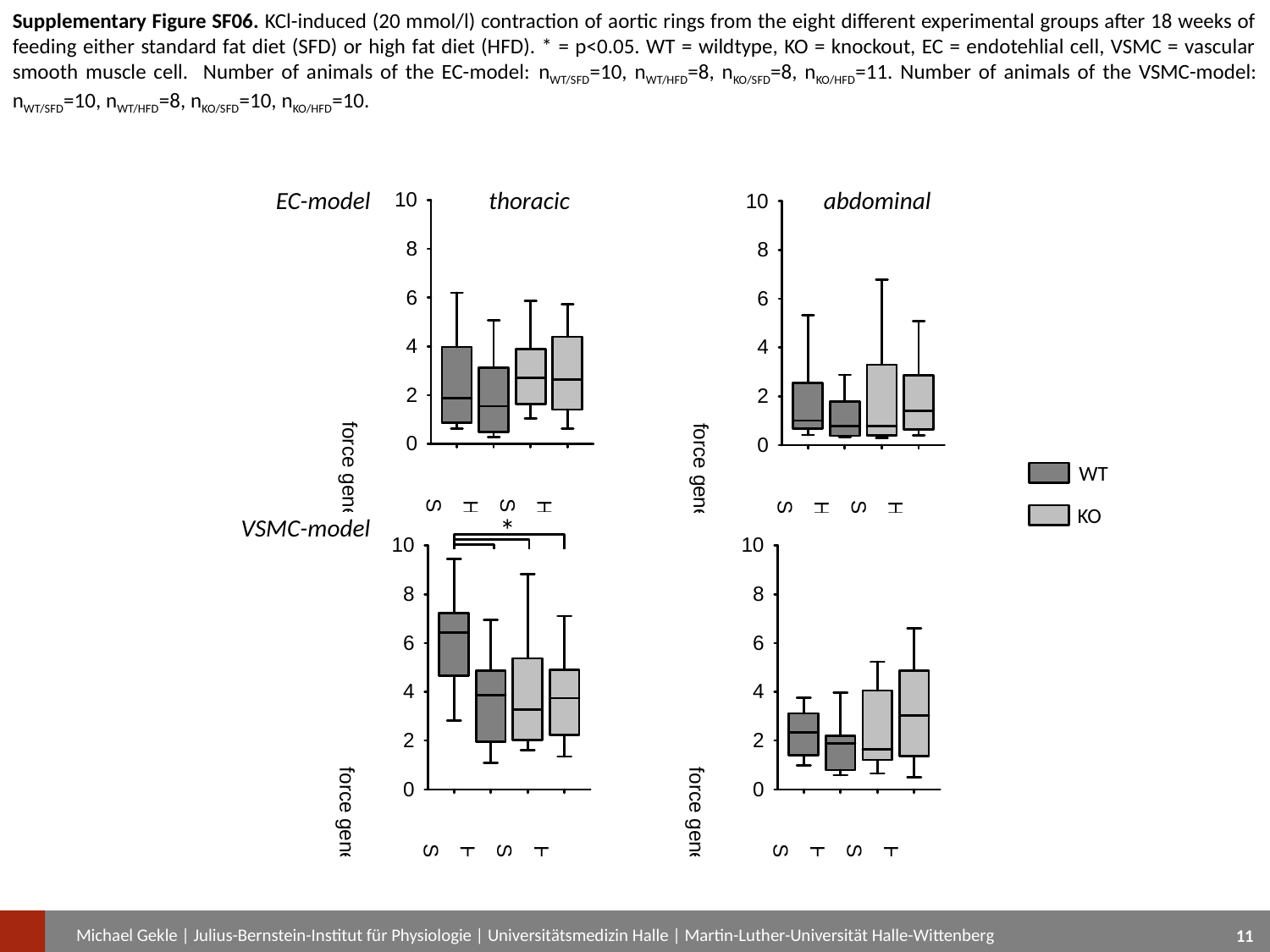

Supplementary Figure SF06. KCl-induced (20 mmol/l) contraction of aortic rings from the eight different experimental groups after 18 weeks of feeding either standard fat diet (SFD) or high fat diet (HFD). * = p<0.05. WT = wildtype, KO = knockout, EC = endotehlial cell, VSMC = vascular smooth muscle cell. Number of animals of the EC-model: nWT/SFD=10, nWT/HFD=8, nKO/SFD=8, nKO/HFD=11. Number of animals of the VSMC-model: nWT/SFD=10, nWT/HFD=8, nKO/SFD=10, nKO/HFD=10.
EC-model
thoracic
abdominal
WT
KO
*
VSMC-model
11

## Slide 12
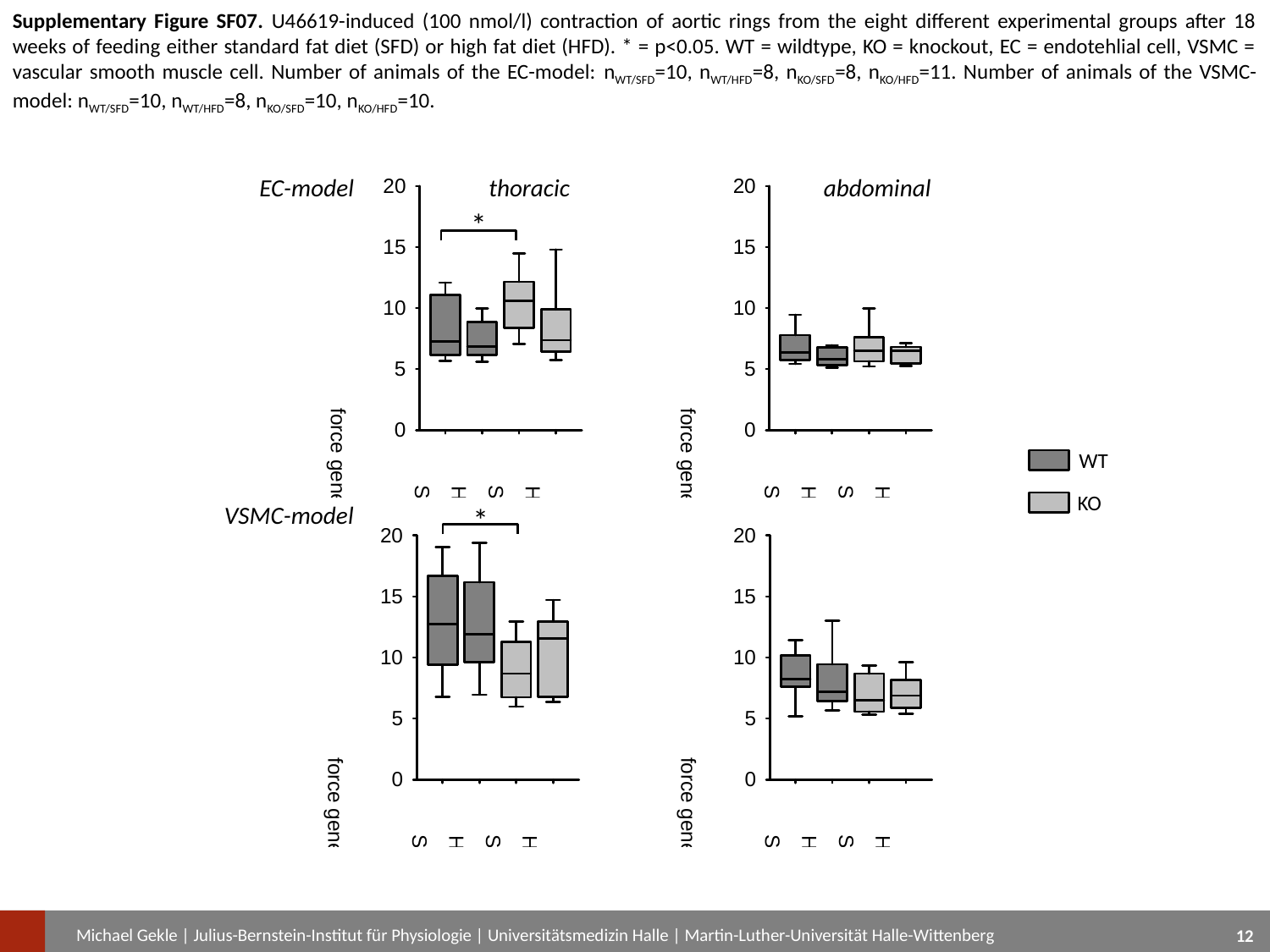

Supplementary Figure SF07. U46619-induced (100 nmol/l) contraction of aortic rings from the eight different experimental groups after 18 weeks of feeding either standard fat diet (SFD) or high fat diet (HFD). * = p<0.05. WT = wildtype, KO = knockout, EC = endotehlial cell, VSMC = vascular smooth muscle cell. Number of animals of the EC-model: nWT/SFD=10, nWT/HFD=8, nKO/SFD=8, nKO/HFD=11. Number of animals of the VSMC-model: nWT/SFD=10, nWT/HFD=8, nKO/SFD=10, nKO/HFD=10.
EC-model
thoracic
abdominal
*
WT
KO
VSMC-model
*
12

## Slide 13
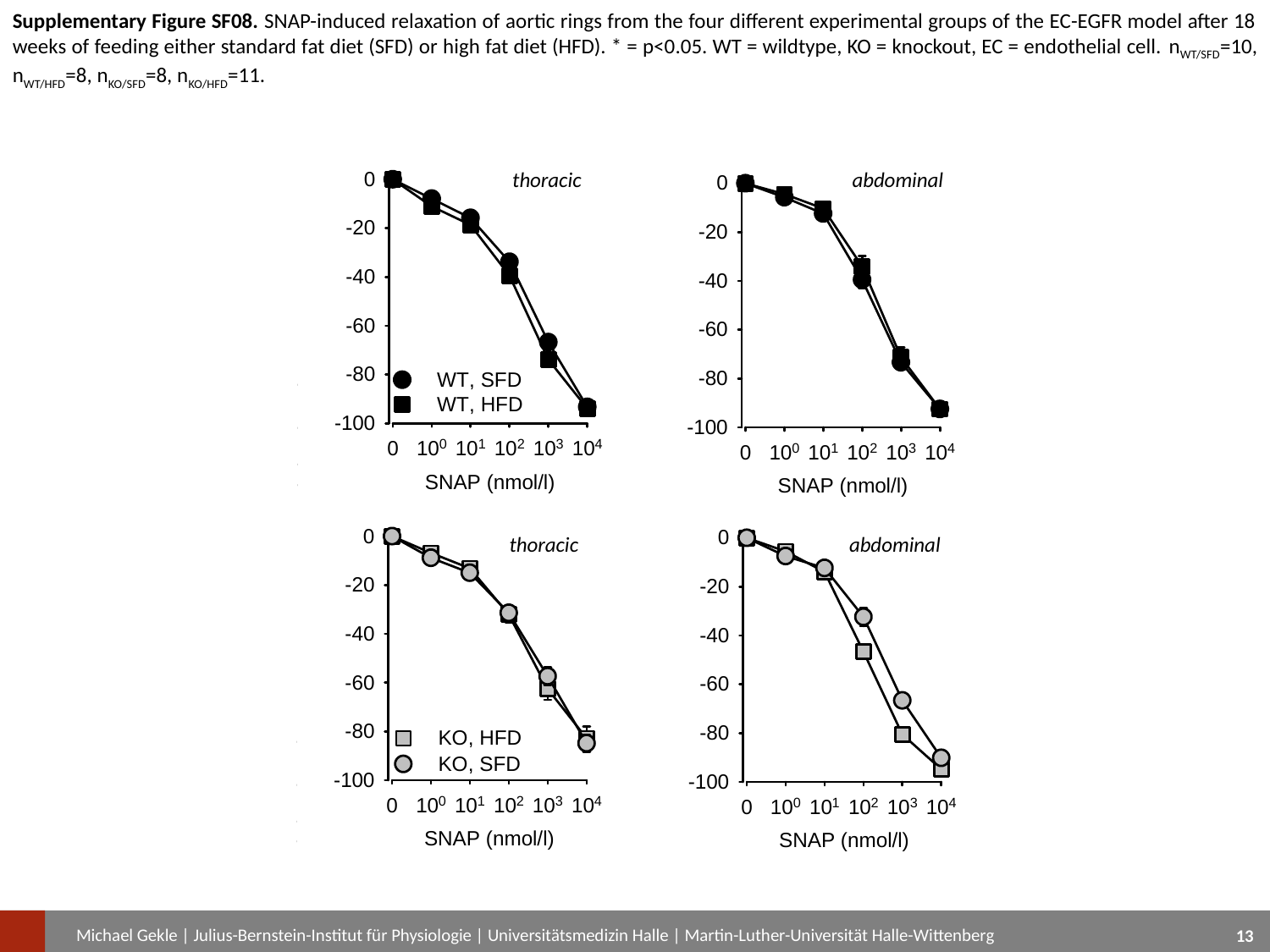

Supplementary Figure SF08. SNAP-induced relaxation of aortic rings from the four different experimental groups of the EC-EGFR model after 18 weeks of feeding either standard fat diet (SFD) or high fat diet (HFD). * = p<0.05. WT = wildtype, KO = knockout, EC = endothelial cell. nWT/SFD=10, nWT/HFD=8, nKO/SFD=8, nKO/HFD=11.
thoracic
abdominal
thoracic
abdominal
13

## Slide 14
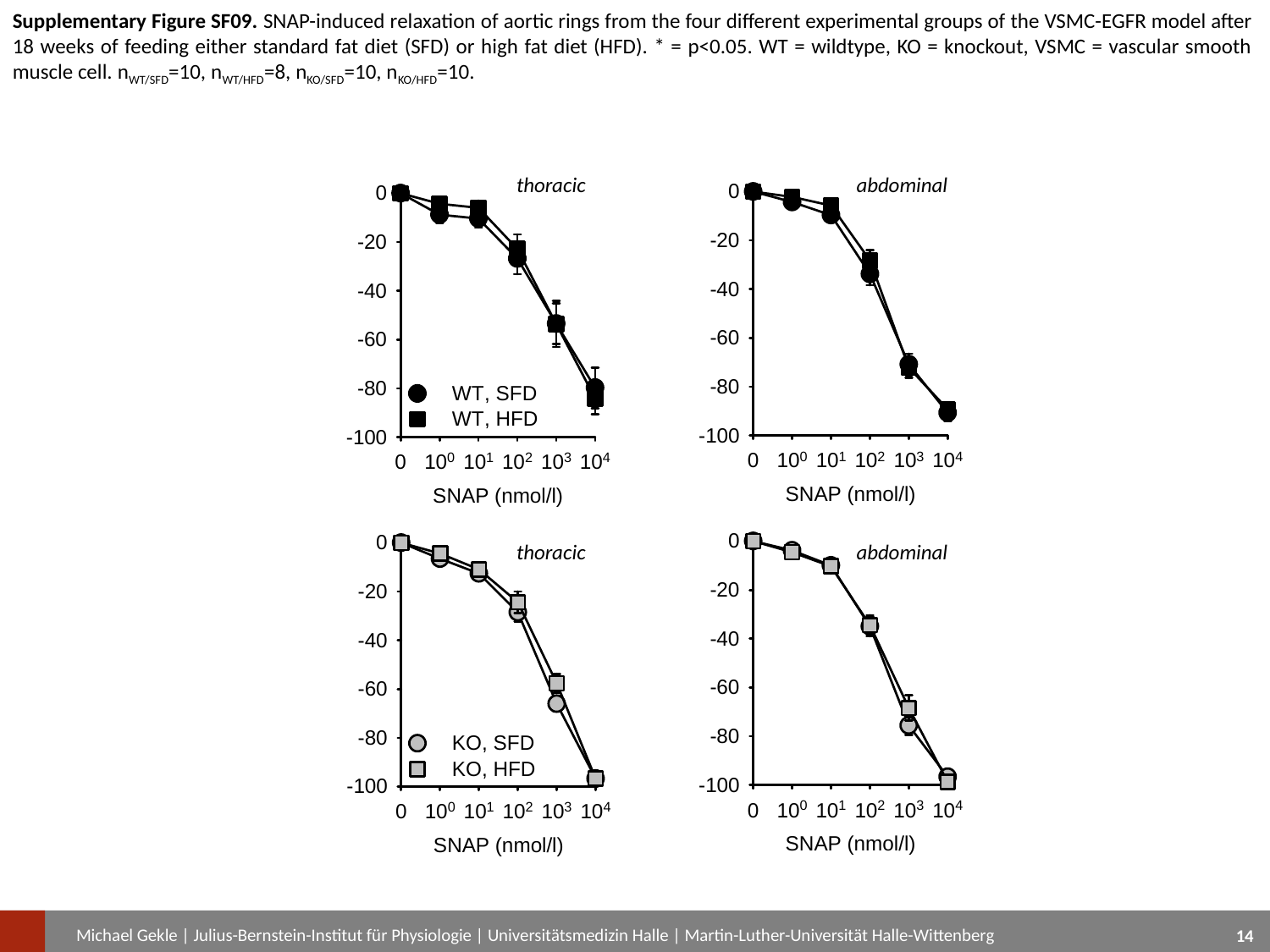

Supplementary Figure SF09. SNAP-induced relaxation of aortic rings from the four different experimental groups of the VSMC-EGFR model after 18 weeks of feeding either standard fat diet (SFD) or high fat diet (HFD). * = p<0.05. WT = wildtype, KO = knockout, VSMC = vascular smooth muscle cell. nWT/SFD=10, nWT/HFD=8, nKO/SFD=10, nKO/HFD=10.
thoracic
abdominal
thoracic
abdominal
14

## Slide 15
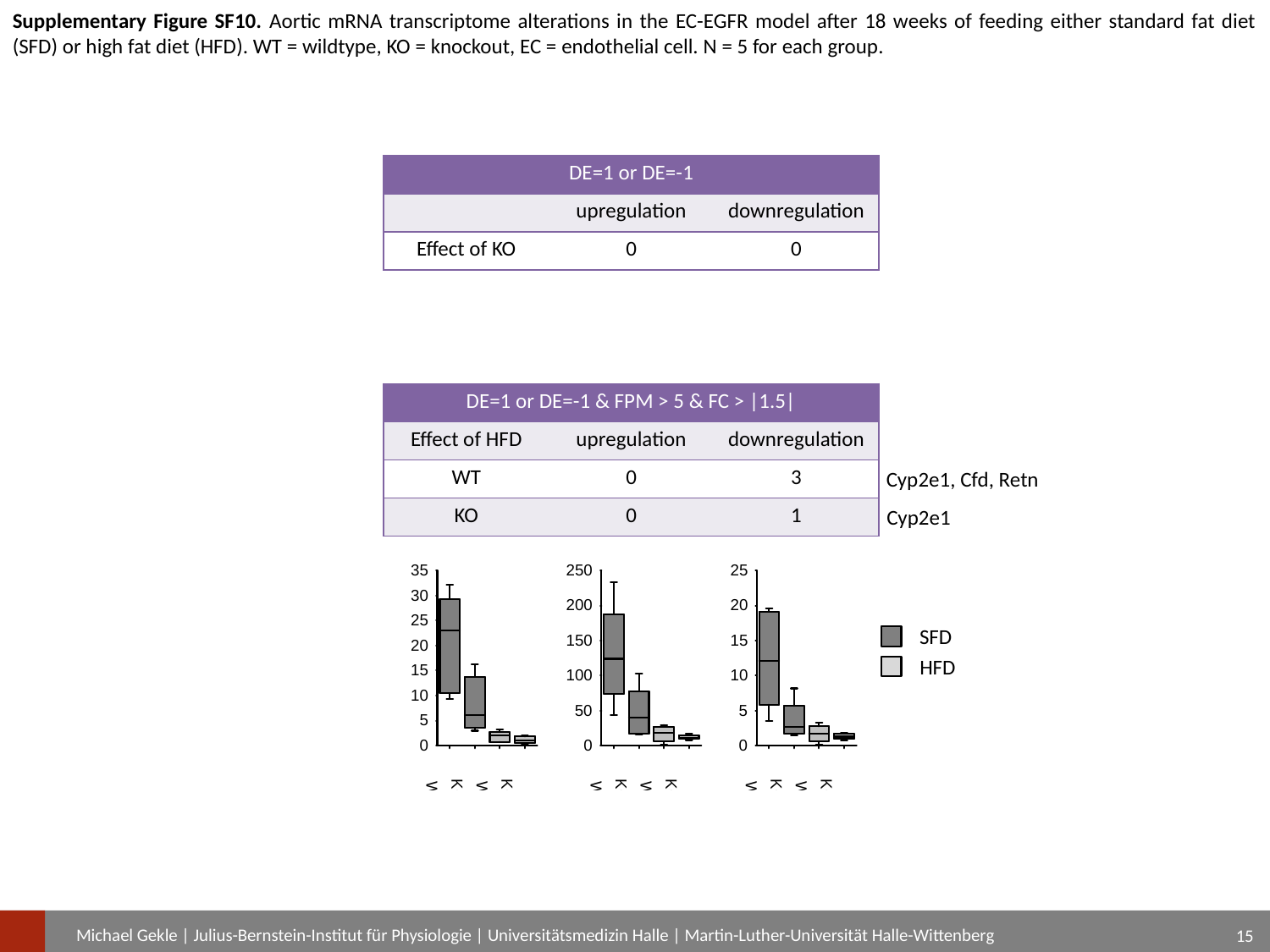

Supplementary Figure SF10. Aortic mRNA transcriptome alterations in the EC-EGFR model after 18 weeks of feeding either standard fat diet (SFD) or high fat diet (HFD). WT = wildtype, KO = knockout, EC = endothelial cell. N = 5 for each group.
| DE=1 or DE=-1 | | |
| --- | --- | --- |
| | upregulation | downregulation |
| Effect of KO | 0 | 0 |
| DE=1 or DE=-1 & FPM > 5 & FC > |1.5| | | |
| --- | --- | --- |
| Effect of HFD | upregulation | downregulation |
| WT | 0 | 3 |
| KO | 0 | 1 |
Cyp2e1, Cfd, Retn
Cyp2e1
SFD
HFD
15

## Slide 16
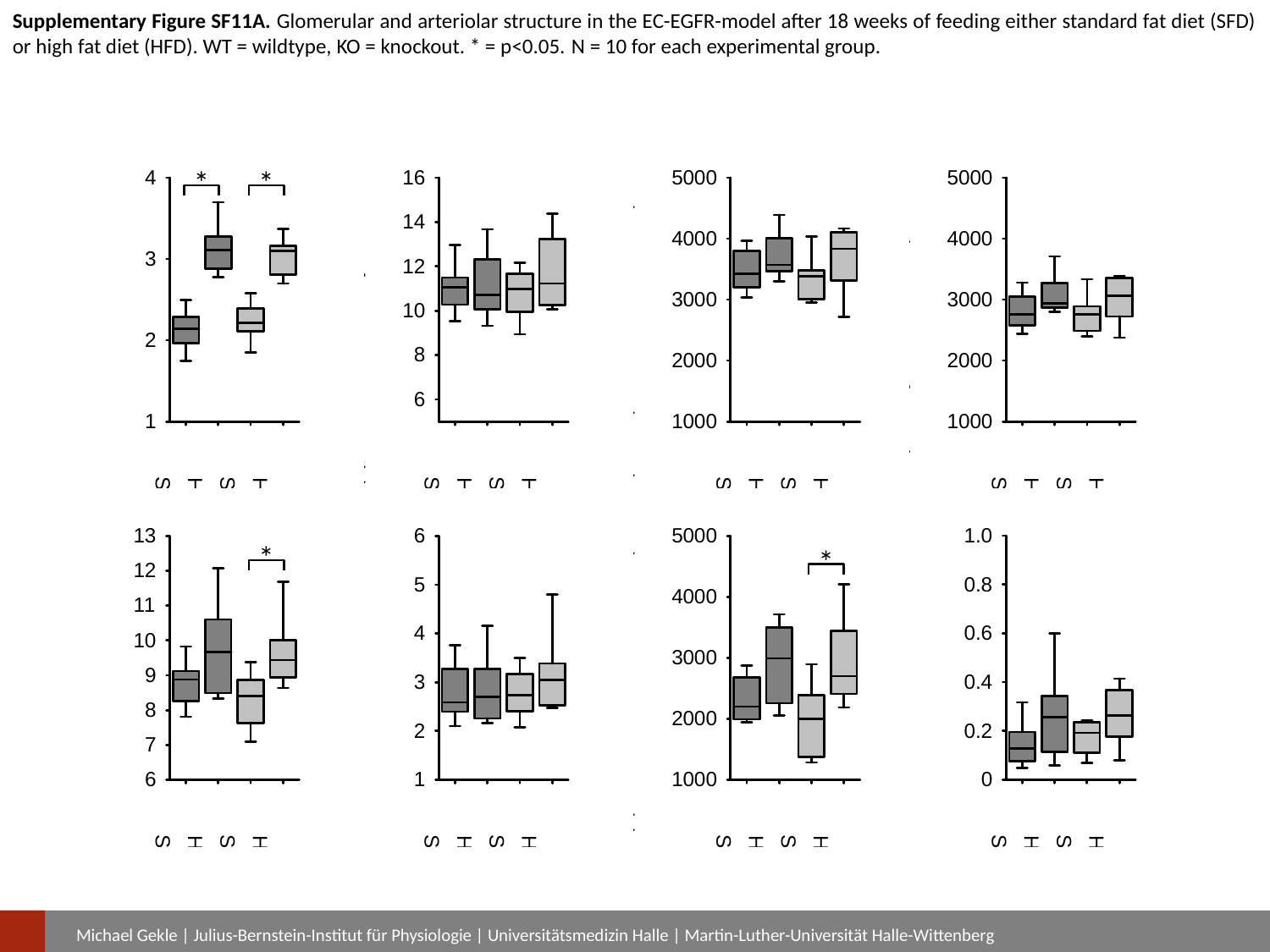

Supplementary Figure SF11A. Glomerular and arteriolar structure in the EC-EGFR-model after 18 weeks of feeding either standard fat diet (SFD) or high fat diet (HFD). WT = wildtype, KO = knockout. * = p<0.05. N = 10 for each experimental group.
*
*
*
*

## Slide 17
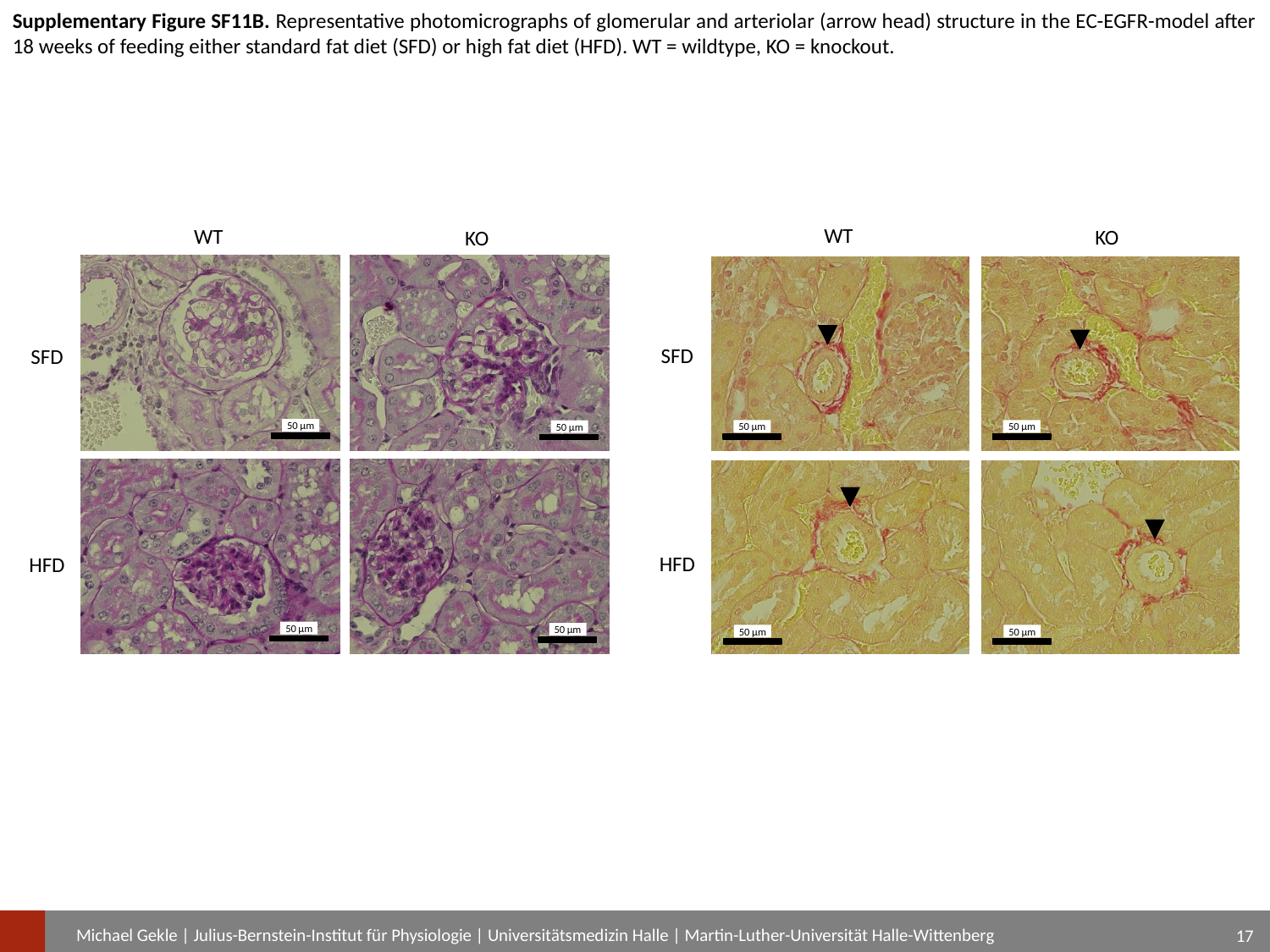

Supplementary Figure SF11B. Representative photomicrographs of glomerular and arteriolar (arrow head) structure in the EC-EGFR-model after 18 weeks of feeding either standard fat diet (SFD) or high fat diet (HFD). WT = wildtype, KO = knockout.
WT
WT
KO
KO
SFD
SFD
50 µm
50 µm
50 µm
50 µm
HFD
HFD
50 µm
50 µm
50 µm
50 µm
17

## Slide 18
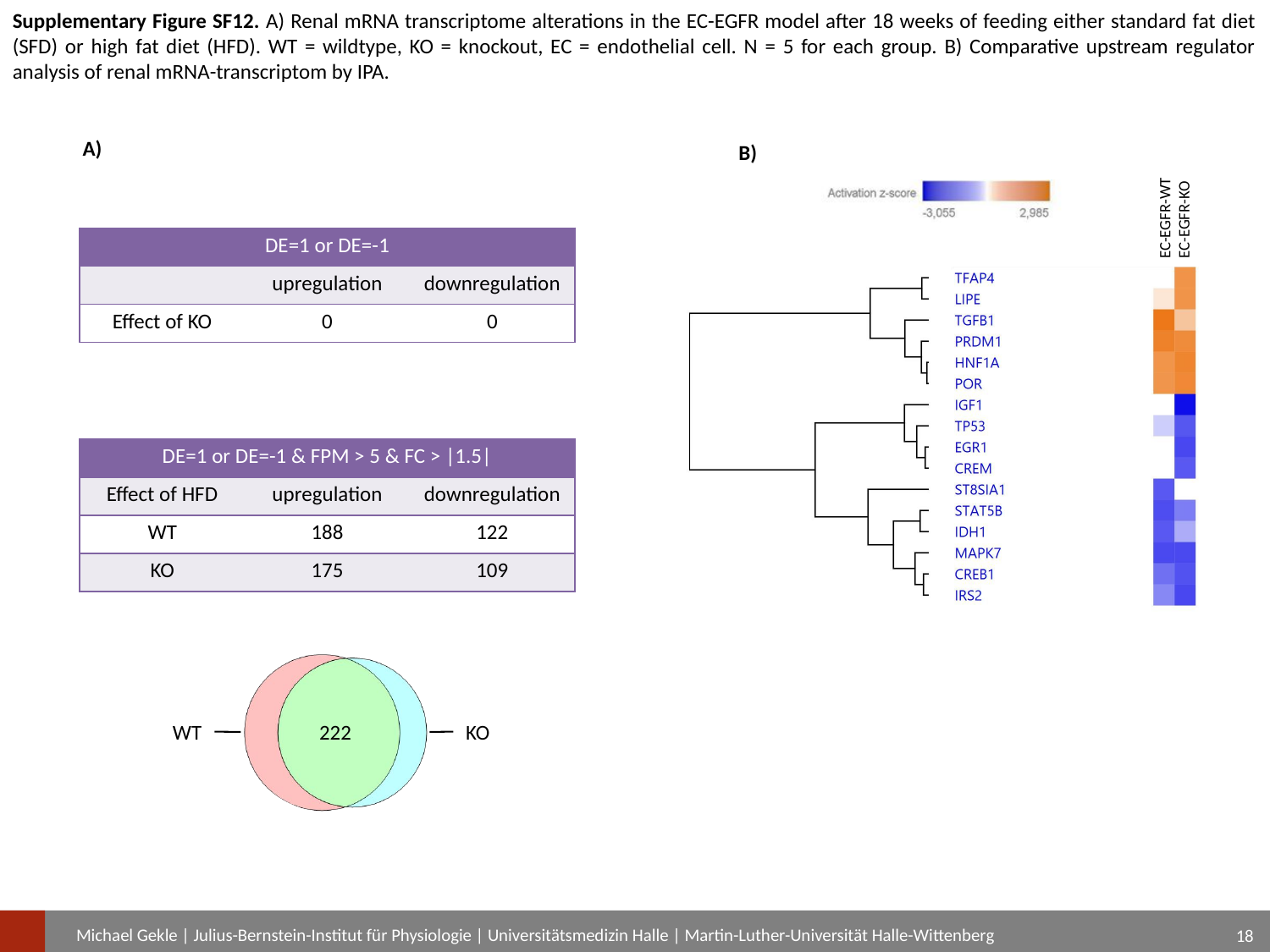

Supplementary Figure SF12. A) Renal mRNA transcriptome alterations in the EC-EGFR model after 18 weeks of feeding either standard fat diet (SFD) or high fat diet (HFD). WT = wildtype, KO = knockout, EC = endothelial cell. N = 5 for each group. B) Comparative upstream regulator analysis of renal mRNA-transcriptom by IPA.
A)
B)
EC-EGFR-WT
EC-EGFR-KO
| DE=1 or DE=-1 | | |
| --- | --- | --- |
| | upregulation | downregulation |
| Effect of KO | 0 | 0 |
| DE=1 or DE=-1 & FPM > 5 & FC > |1.5| | | |
| --- | --- | --- |
| Effect of HFD | upregulation | downregulation |
| WT | 188 | 122 |
| KO | 175 | 109 |
WT
222
KO
18

## Slide 19
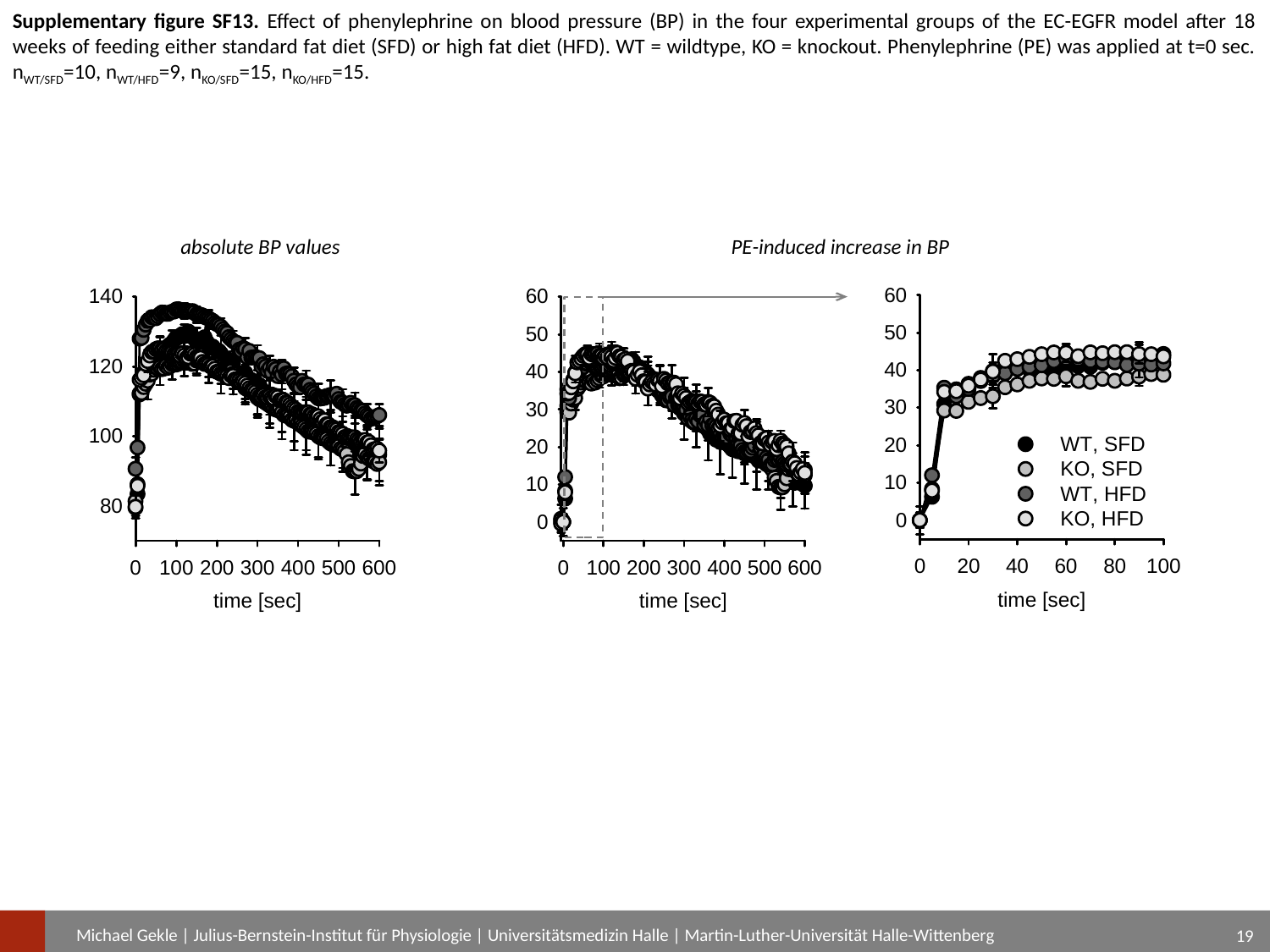

Supplementary figure SF13. Effect of phenylephrine on blood pressure (BP) in the four experimental groups of the EC-EGFR model after 18 weeks of feeding either standard fat diet (SFD) or high fat diet (HFD). WT = wildtype, KO = knockout. Phenylephrine (PE) was applied at t=0 sec. nWT/SFD=10, nWT/HFD=9, nKO/SFD=15, nKO/HFD=15.
absolute BP values
PE-induced increase in BP
19

## Slide 20
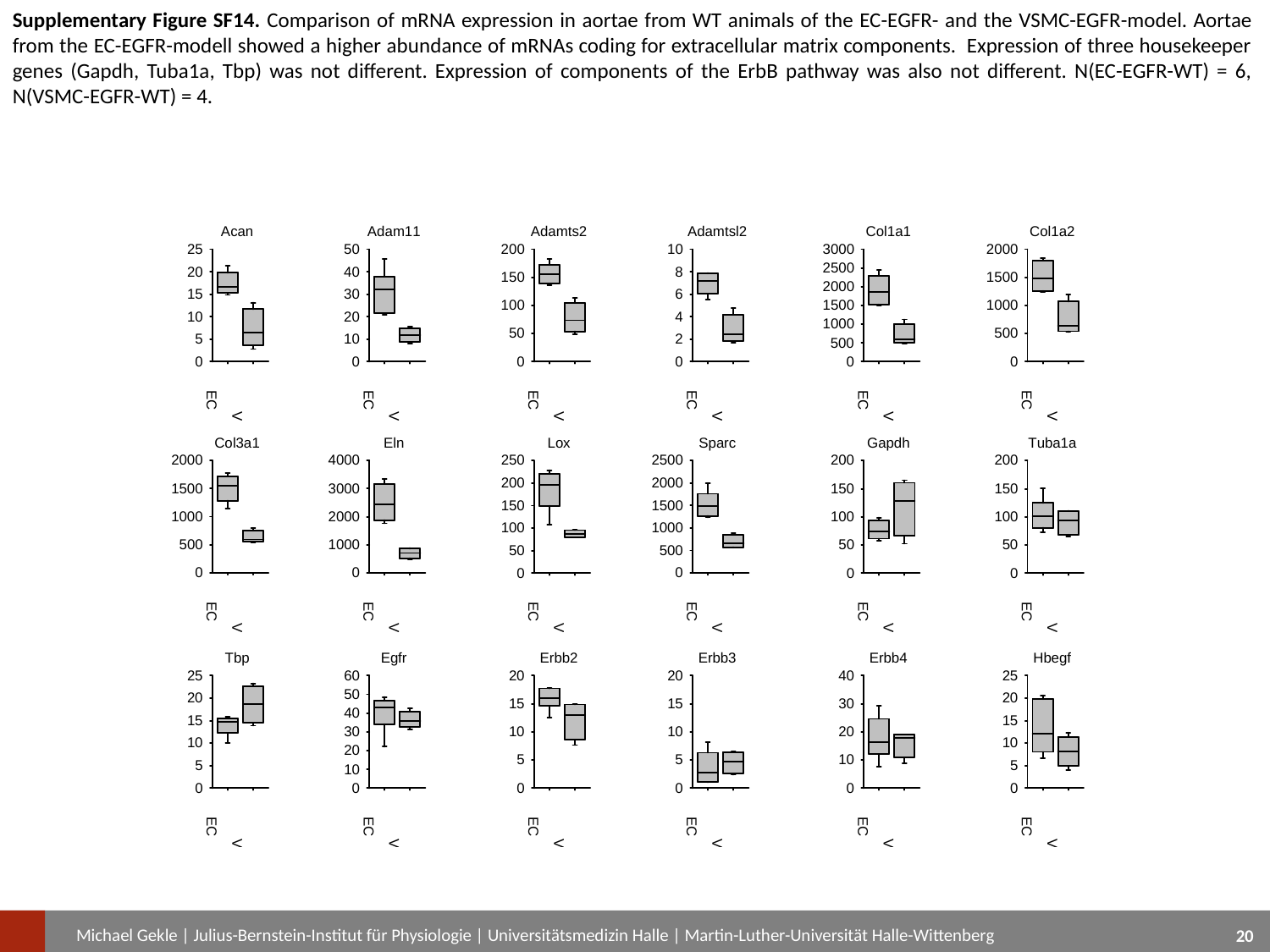

Supplementary Figure SF14. Comparison of mRNA expression in aortae from WT animals of the EC-EGFR- and the VSMC-EGFR-model. Aortae from the EC-EGFR-modell showed a higher abundance of mRNAs coding for extracellular matrix components. Expression of three housekeeper genes (Gapdh, Tuba1a, Tbp) was not different. Expression of components of the ErbB pathway was also not different. N(EC-EGFR-WT) = 6, N(VSMC-EGFR-WT) = 4.
20

## Slide 21
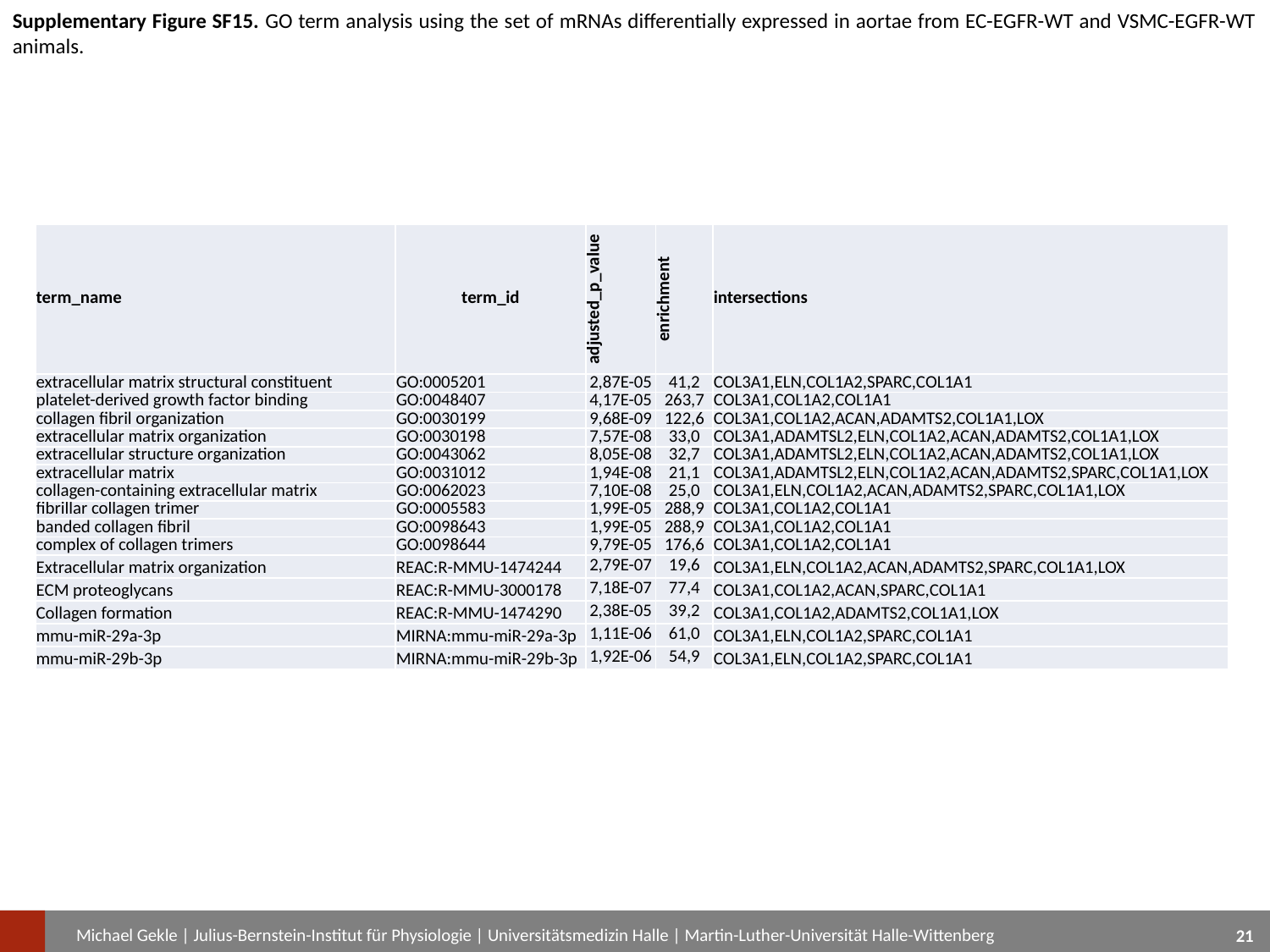

Supplementary Figure SF15. GO term analysis using the set of mRNAs differentially expressed in aortae from EC-EGFR-WT and VSMC-EGFR-WT animals.
| term\_name | term\_id | adjusted\_p\_value | enrichment | intersections |
| --- | --- | --- | --- | --- |
| extracellular matrix structural constituent | GO:0005201 | 2,87E-05 | 41,2 | COL3A1,ELN,COL1A2,SPARC,COL1A1 |
| platelet-derived growth factor binding | GO:0048407 | 4,17E-05 | 263,7 | COL3A1,COL1A2,COL1A1 |
| collagen fibril organization | GO:0030199 | 9,68E-09 | 122,6 | COL3A1,COL1A2,ACAN,ADAMTS2,COL1A1,LOX |
| extracellular matrix organization | GO:0030198 | 7,57E-08 | 33,0 | COL3A1,ADAMTSL2,ELN,COL1A2,ACAN,ADAMTS2,COL1A1,LOX |
| extracellular structure organization | GO:0043062 | 8,05E-08 | 32,7 | COL3A1,ADAMTSL2,ELN,COL1A2,ACAN,ADAMTS2,COL1A1,LOX |
| extracellular matrix | GO:0031012 | 1,94E-08 | 21,1 | COL3A1,ADAMTSL2,ELN,COL1A2,ACAN,ADAMTS2,SPARC,COL1A1,LOX |
| collagen-containing extracellular matrix | GO:0062023 | 7,10E-08 | 25,0 | COL3A1,ELN,COL1A2,ACAN,ADAMTS2,SPARC,COL1A1,LOX |
| fibrillar collagen trimer | GO:0005583 | 1,99E-05 | 288,9 | COL3A1,COL1A2,COL1A1 |
| banded collagen fibril | GO:0098643 | 1,99E-05 | 288,9 | COL3A1,COL1A2,COL1A1 |
| complex of collagen trimers | GO:0098644 | 9,79E-05 | 176,6 | COL3A1,COL1A2,COL1A1 |
| Extracellular matrix organization | REAC:R-MMU-1474244 | 2,79E-07 | 19,6 | COL3A1,ELN,COL1A2,ACAN,ADAMTS2,SPARC,COL1A1,LOX |
| ECM proteoglycans | REAC:R-MMU-3000178 | 7,18E-07 | 77,4 | COL3A1,COL1A2,ACAN,SPARC,COL1A1 |
| Collagen formation | REAC:R-MMU-1474290 | 2,38E-05 | 39,2 | COL3A1,COL1A2,ADAMTS2,COL1A1,LOX |
| mmu-miR-29a-3p | MIRNA:mmu-miR-29a-3p | 1,11E-06 | 61,0 | COL3A1,ELN,COL1A2,SPARC,COL1A1 |
| mmu-miR-29b-3p | MIRNA:mmu-miR-29b-3p | 1,92E-06 | 54,9 | COL3A1,ELN,COL1A2,SPARC,COL1A1 |
21
